# Nitric oxide mediates nitrate induced alleviation of waterlogging stress in cucumber

**DOI:** 10.1038/s41598-025-00321-x

**Published:** 2025-05-01

**Authors:** Neda Hesari, Iman Mirmazloum, Katalin Jäger, Henriett Kolozs, Erzsébet Kiss-Bába, Maria Eduarda Soares Ramos, Imran Khan, Dorina Babinyec-Czifra, Anita Szegő, István Papp

**Affiliations:** 1 Department of Plant Physiology and Plant Ecology, Institute of Agronomy, Hungarian University of Agriculture and Life Sciences, Budapest, Hungary; 2 Biological Resources Department, HUN-REN Centre for Agricultural Research, Martonvásár, Hungary; 3 Doctoral School of Biology and Institute of Biology, ELTE Eötvös Loránd University, Budapest, Hungary

**Keywords:** Waterlogging, Nitrate, Nitric oxide, Cucumber, Plant molecular biology, Plant stress responses

## Abstract

Effects of waterlogging (WL) stress and its mitigation by nitrate (NO_3_^–^) were investigated in a commercial cucumber hybrid line. WL resulted in a shift toward anaerobic metabolism in roots and development of adventitious roots (ARs). In WL stressed roots exodermal, extracellular H_2_O_2_ accumulation and protein nitration were detected. Shoot growth was retarded with signs of oxidative stress. Nitrate content decreased in both root and shoot. In the root WL upregulated several genes implicated in hypoxic response, including transcripts of *CsRAP2.3*,* CsRBOH*s and the *CsHem3* phytoglobin genes. Nitrate supplementation restored shoot growth and mitigated oxidative stress. Fermentative metabolism was reduced in the roots. Nitrate levels increased substantially, expression of *NRT1* genes was induced, supporting improved NO_3_^−^ uptake and delivery. According to chemical analysis and fluorescent microscopy NO accumulated in the vascular cylinder of roots. NO depletion by cPTIO confirmed a role for NO in stress mitigation. cPTIO treatment decreased AR formation and foliar nitrate levels. It decreased protein nitration and strongly downregulated nitrate transporters’ gene expression in roots. Overall, our work revealed nitrate triggered, NO mediated alleviation of WL stress in cucumber. Resolution of WL induced energy crisis in the roots by nitrate treatment was attributed to parallel effects of anaerobic respiration and AR formation. Shoot growth repression was probably alleviated by improved root functions, including elevated nitrate transport. Expression of *NRT1* nitrate transporter genes was induced by nitrate, and this induction was found NO dependent in WL stressed cucumber.

**Supplementary Information:**

The online version contains supplementary material available at 10.1038/s41598-025-00321-x.

## Introduction

Nitrogen is the most represented macroelement in plants, its major form in aerated soils is nitrate. Nitrate also has an established regulatory function as signaling molecule, affecting gene expression and growth^1,2^. It regulates both short- and long-term responses at the gene expression level by initiating a well-defined signaling pathway^3^. Studies so far have concentrated more on low nitrate availability, as field crops often face that challenge. However, high nitrate supply is also of paramount importance in intensive agricultural systems as it is a key determinant of yield and represents a source of environmental pollution by leaching from excessive fertigation^4^.

Hypoxia is a fundamental stress condition, resulting in growth retardation among other symptoms in a number of plant species. Waterlogging (WL) and submergence are the most frequently studied situations where hypoxia may develop^5^. WL stress should be considered in soilless vegetable cultivation where root growth is restricted to a confined space, and intensive fertigation is applied^6^. As a model, cucumber seems particularly suited for studying mechanisms of WL stress responses, as hybrids of this species showed different, but generally high tolerance levels, especially under nutrient rich conditions^6^. In other studies cucumber genotypes also displayed variable tolerance against WL conditions^7,8^.

In WL stress, due to changes of energy balance and pH relations, repressed root hydraulic conductivity and compromised aquaporin function are known to inhibit xylem transport^9^ creating osmotic stress in shoot. Shoot growth inhibition has been regarded as a consequence of the osmotic stress derived from diminished root functions^10^. Hormonal effects, especially perturbed abscisic acid/gibberellic acid homeostasis may contribute to growth retardation^11^.

In hypoxia, responsive genes, like those coding for ADH enzymes, are orchestrated by the ERFVII transcription factor (TF) family^12^. These TFs may get activated post-translationally in hypoxia, pending on processing a canonical N terminal amino acid sequence, defined by the N end rule. Low energy status of the cells (low ATP level) can also trigger the ERFVII activation pathway^13,14^.

**Fig. 1:**
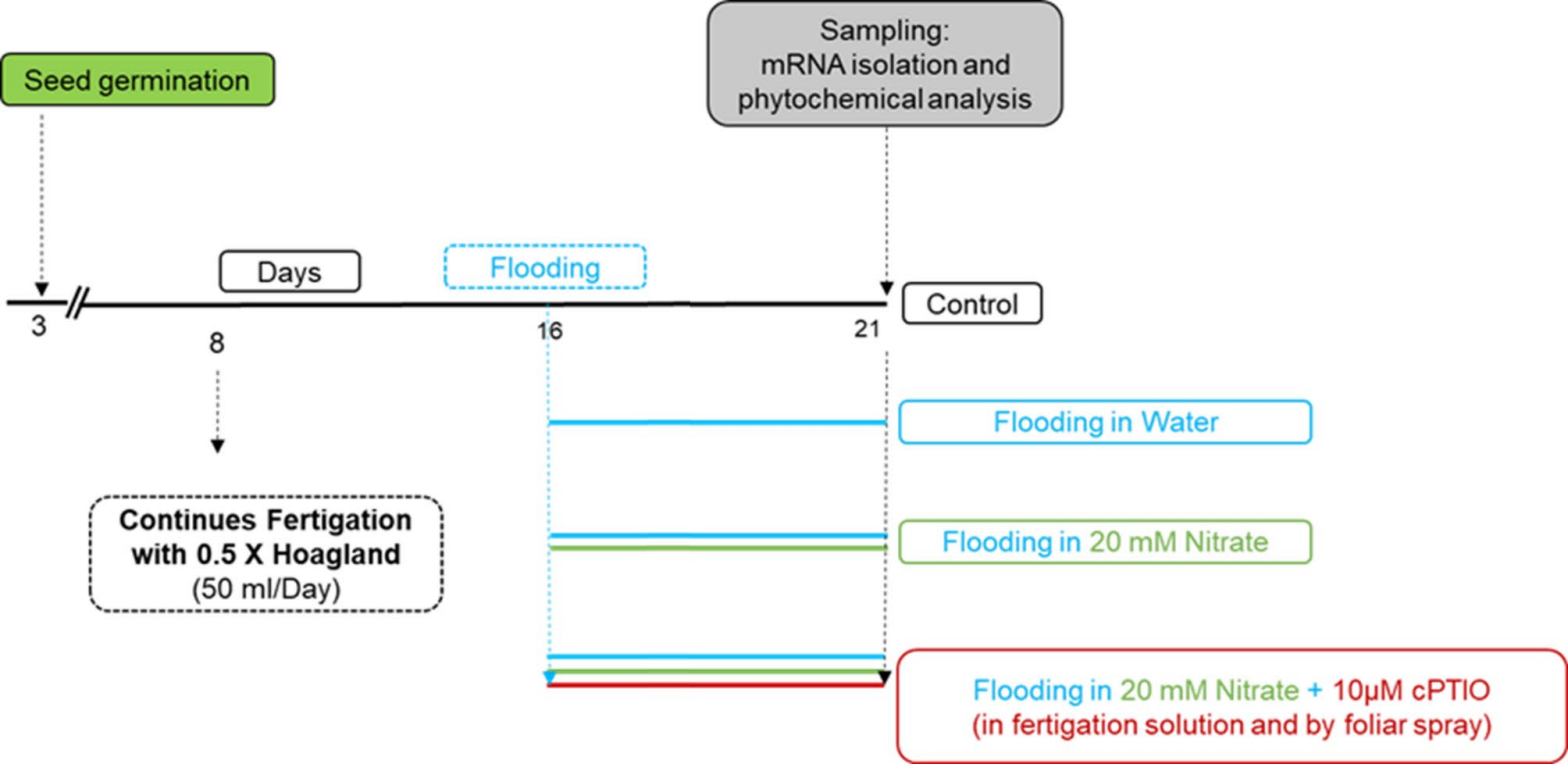
Schematic illustration of the experimental design and treatments of cucumber seedlings.

Nitric oxide (NO) is a free radical with a signaling role in both animals and plants^15,16^. As a gaso-transmitter it has been found involved in modulating divers stress responses, in many cases exerting mitigating effects in abiotic and biotic stresses^17,18^. This signaling molecule may be synthesized in several ways in plants, including several oxidative and reductive pathways. Reductive pathways include the reduction of nitrate ions by nitrate reductase (NRase) activity^19–21^. The phytoglobin/NO (Pgb/NO) cycle is a way of scavenging NO from the cytoplasm^22^. Enzyme activity of S-nitroso-glutathione reductases (GSNOR) leads to decomposition of S-nitroso-glutathione, ultimately also resulting in declining NO levels^23^. In hypoxic tissues the nitrite generated by NRase enters the mitochondria either by diffusion or by an unknown transporter. In the matrix nitrite serves as electron acceptor at probably more than one component of the electron transport chain (mETC), which reduces it to NO^15^ in the process of anaerobic respiration. These events can improve the energy supply of the flooded roots. However, high NO levels may feed-back inhibit essential components of this same process, such as NRase and mETC^20,24^. In the present study effects of nitrate supplemented media were studied in consequences of WL stress on a commercial cucumber hybrid line. Shoot growth, nitrate metabolism and transport as well as redox status were investigated, also including some potential signaling components. Involvement of NO as a potential modulator of stress responses was also tested by the application of a NO scavenger (Fig. 1).

## Results

### Confirmation of hypoxia and the effects of waterlogging stress on plant growth

**Fig. 2:**
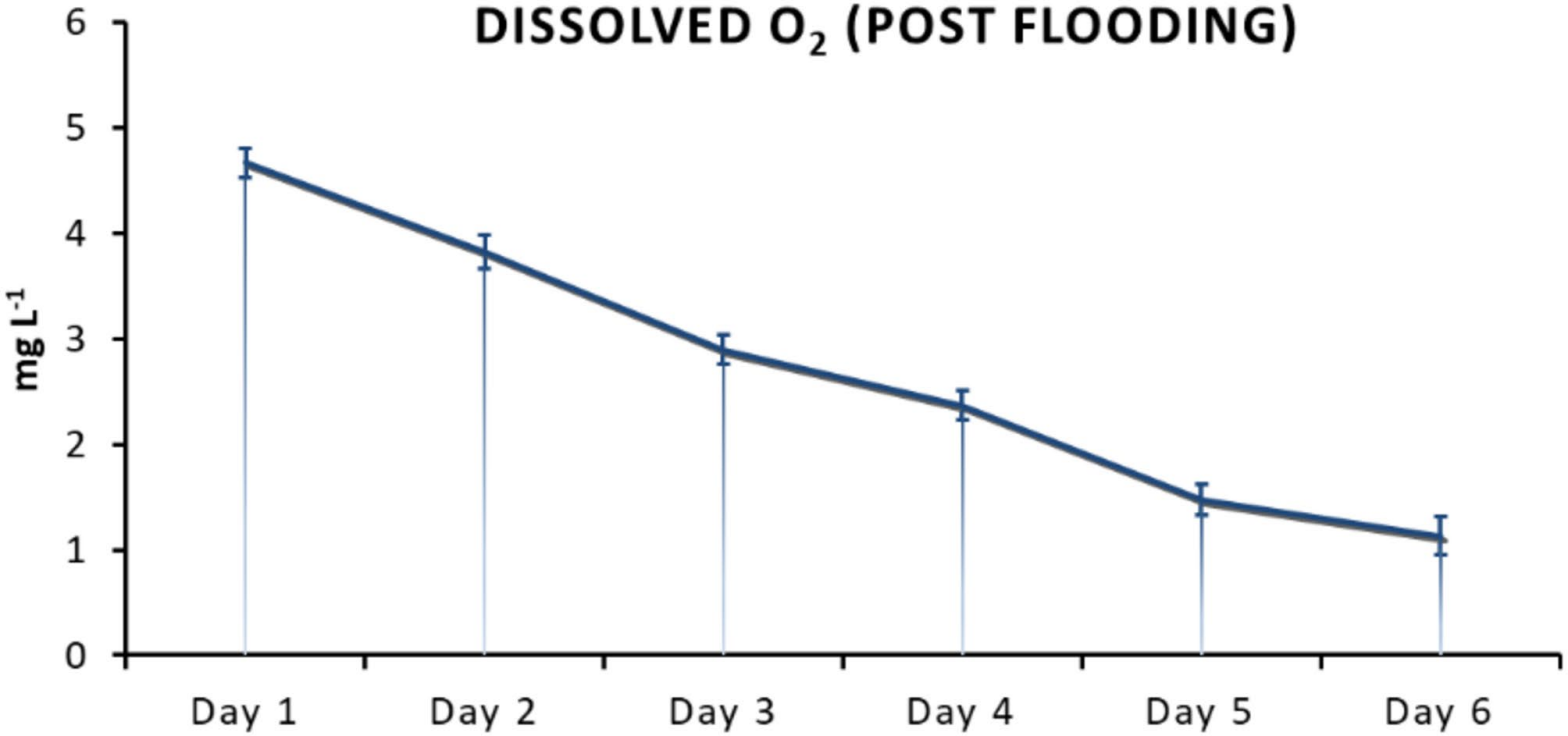
Dissolved oxygen concentration in the waterlogging solutions. Data were recorded for 6 days by a using portable oxygen-measuring device (Voltcraft DO-101) to monitor the extent of hypoxia around the roots of the young seedlings of control and treated cucumber plants.

To ensure that the plants experienced hypoxia, the actual dissolved oxygen concentration was constantly measured in the waterlogging solutions every day. As shown in Fig. 2, the dissolved oxygen level decreased in the media in all treatments, showing no discernable variation in dynamics. By the end of the treatments, low oxygen levels in the media progressively limited O_2_ availability to the roots.

**Fig. 3:**
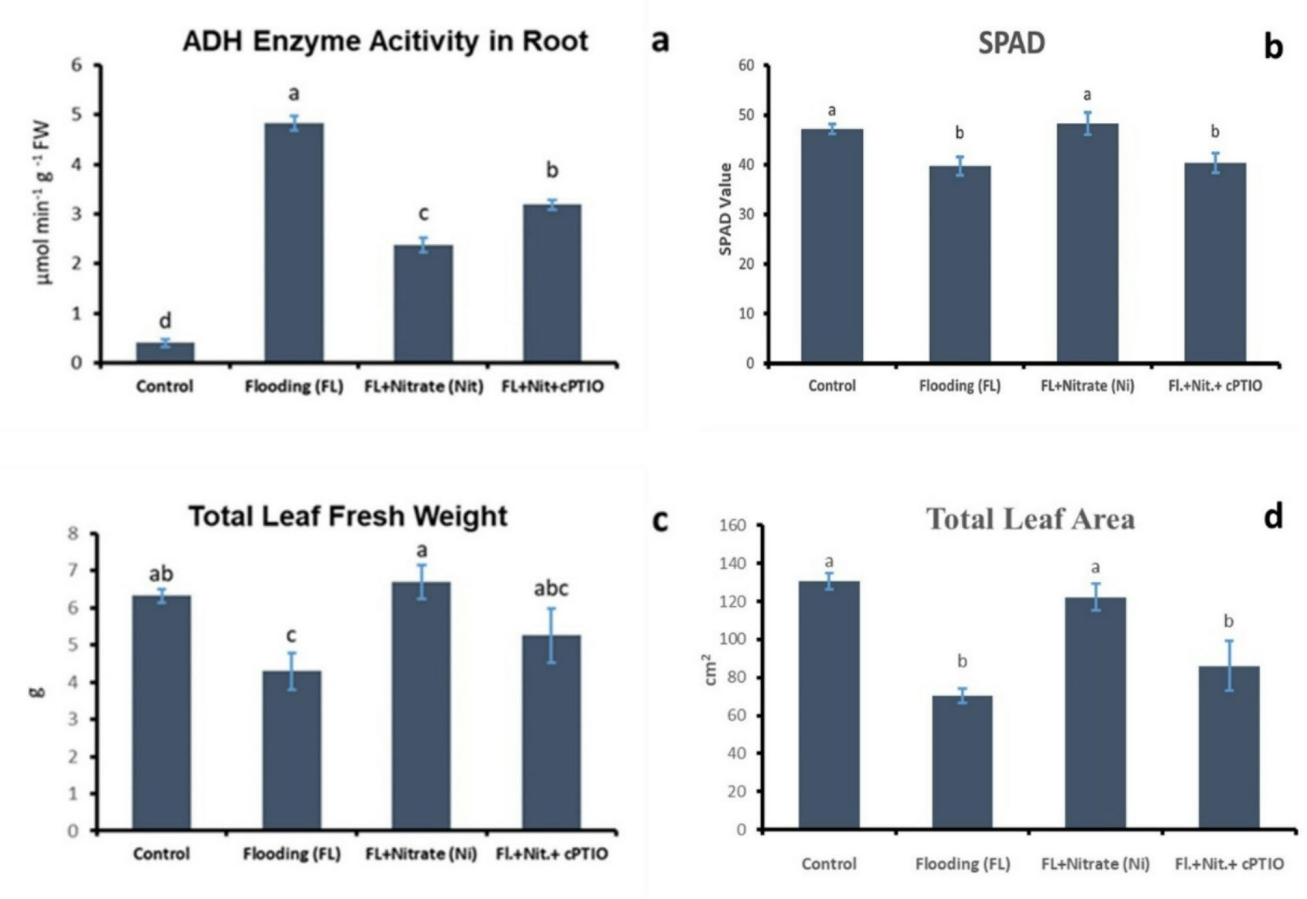
ADH enzyme activity (a), SPAD values (b), leaf fresh weight (c), and total leaf area (d) of control and waterlogged cucumber seedlings treated with nitrate (20 mM), and with nitrate plus cPTIO. Different letters on bars mark significant changes at 95% (*p* < 0.05) based on the Fisher’s least significant difference (LSD) test, used for comparison of the mean values.

**Fig. 4:**
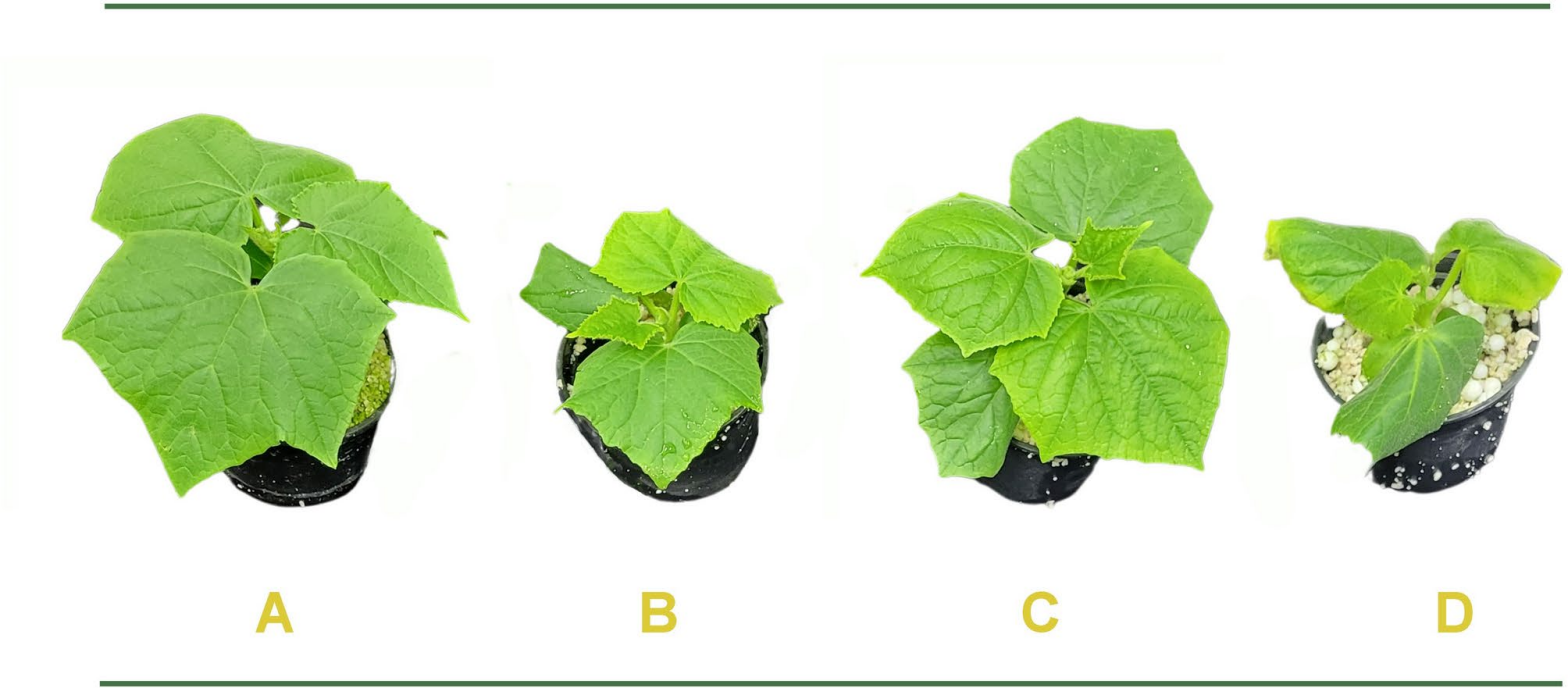
Representative images of cucumber seedlings grown under control conditions (A), waterlogging stress (B), waterlogging stress with 20 mM nitrate (C) and waterlogging stress with 20 mM nitrate plus cPTIO (D). Plants were grown in rockwool cubes placed in perlite, as described in the section Methods. Differential growth is quantified as illustrated in Fig. 3c and d.

**Fig. 5:**
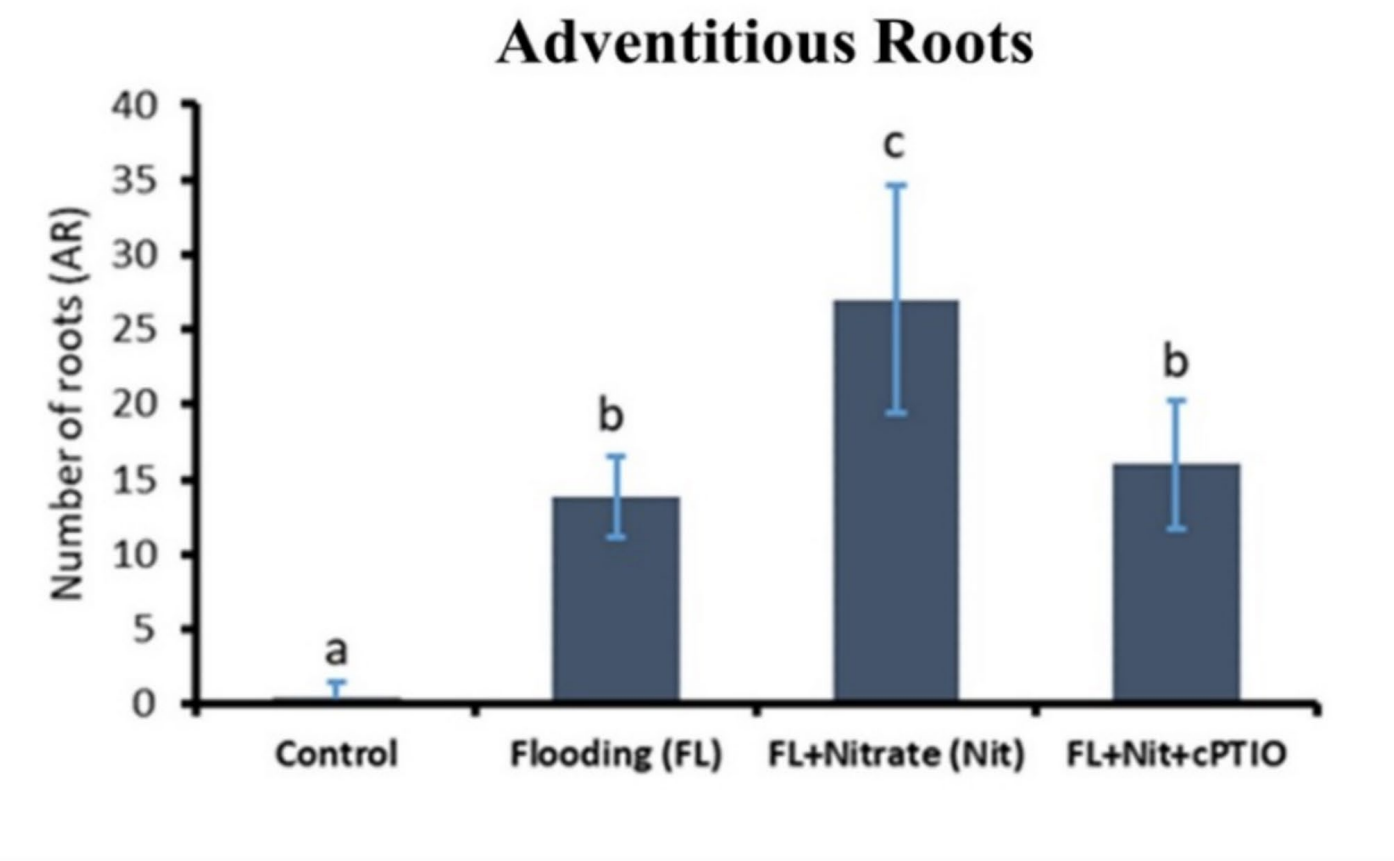
Adventitious root numbers of control and waterlogged cucumber seedlings treated with nitrate (20 mM), and with nitrate plus cPTIO. Different letters on bars mark significant changes at 95% (*p* < 0.05) based on the Fisher’s least significant difference (LSD) test, used for comparison of the mean values. Labels and abbreviations are the same as in Fig. 3.

Alcohol dehydrogenase activity is an indicator of fermentative metabolism triggered by hypoxic conditions. This indicator showed strong induction of fermentation in WL treated roots, testifying hypoxic stress (Fig. 3a). This was partly reverted by parallel nitrate application, while ADH activity was again partially induced by the WL plus nitrate plus cPTIO treatment (Fig. 3a). SPAD values, that reflect chlorophyll content of leaves, significantly decreased (*p* < 0.05) after the WL treatment, but nitrate supplementation increased the SPAD values. WL plus nitrate plus cPTIO treatment acted in the reverse direction, repressing the values to the level of flooded plants (Fig. 3b). WL treatment significantly decreased (*p* < 0.05) shoot growth (shoot fresh weight and total leaf area), while providing additional nitrate (20 mM) effectively mitigated this effect (Figs. 3c-d). On the other hand, cPTIO application alongside the 20 mM nitrate reversed the mitigating effect of nitrate. The effect of waterlogging stress and the application of 20 mM nitrate and 20 mM nitrate + cPTIO on cucumber seedlings are presented by representative images in Fig. 4. WL induced AR formation, what was further promoted by nitrate, and partly reverted by cPTIO treatments (Fig. 5).

Labels and abbreviations for treatments correspond to their mentioning in the text as follows: Flooding (FL) – WL; FL + Nitrate (Nit) – WL/NO_3_^−^; FL + Nit + cPTIO – WL/NO_3_^−^/cPTIO.

**Fig. 6:**
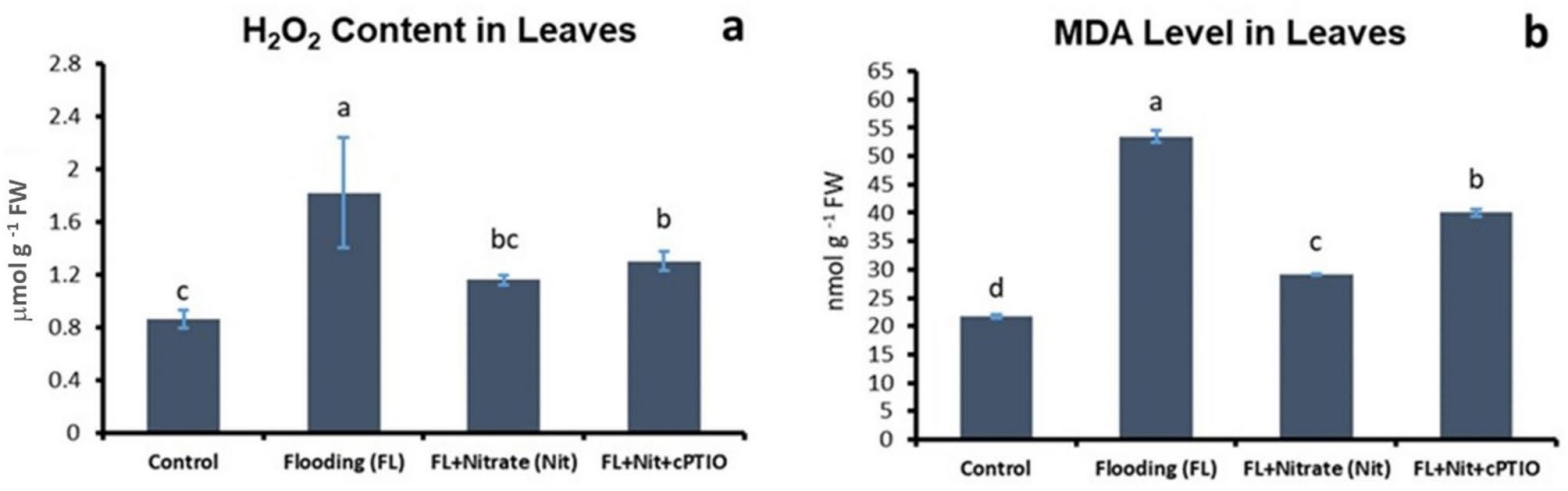
Hydrogen peroxide (a) and malondialdehyde (MDA) concentrations (b) in leaves of control and waterlogged cucumber seedlings treated with nitrate (20 mM), and with nitrate plus cPTIO. Different letters on bars mark significant changes at 95% (*p* < 0.05) based on the least significant difference (LSD) test, used for comparison of the mean values. Labels and abbreviations are the same as in Fig. 3.

Foliar levels of H_2_O_2_ were significantly higher (*p* < 0.05) in plants of WL treatment, which was partially reversed by nitrate application (Fig. 6a), indicating the positive effect of the applied nitrate treatment. Although, a slight increase in H_2_O_2_ was observed in leaves after parallel treatments with nitrate plus cPTIO, under flooding condition, but the change was not significant. The level of malondialdehyde (MDA); an indicator of lipid peroxidation subsequent to stress, was also changed significantly with its highest level being detected in plants under flooding stress (Fig. 6b). Similar to the H_2_O_2_ pattern and as expected, nitrate supplementation significantly reduced (*p* < 0.05) the MDA level in leaves while, when nitrate was supplied with cPTIO, the mitigation effect was less pronounced but still significantly lower than those in WL treatment. The interfering effect of cPTIO on nitrate-induced mitigation of lipid peroxidation was confirmed by changes of MDA level.

Foliar levels of H_2_O_2_ increased with WL treatment, which was partially reversed by nitrate application and further decreased by nitrate plus cPTIO.

The SPAD values, which reflect the chlorophyll content of leaves, followed the same trend, i.e., WL stress decreased, nitrate increased the value, while nitrate plus cPTIO acted in the reverse direction compared to nitrate.

### Mitigating effects of nitrate and nitrate-derived NO

#### Nitrate content, nitrate reductase activity and NO level

**Fig. 7:**
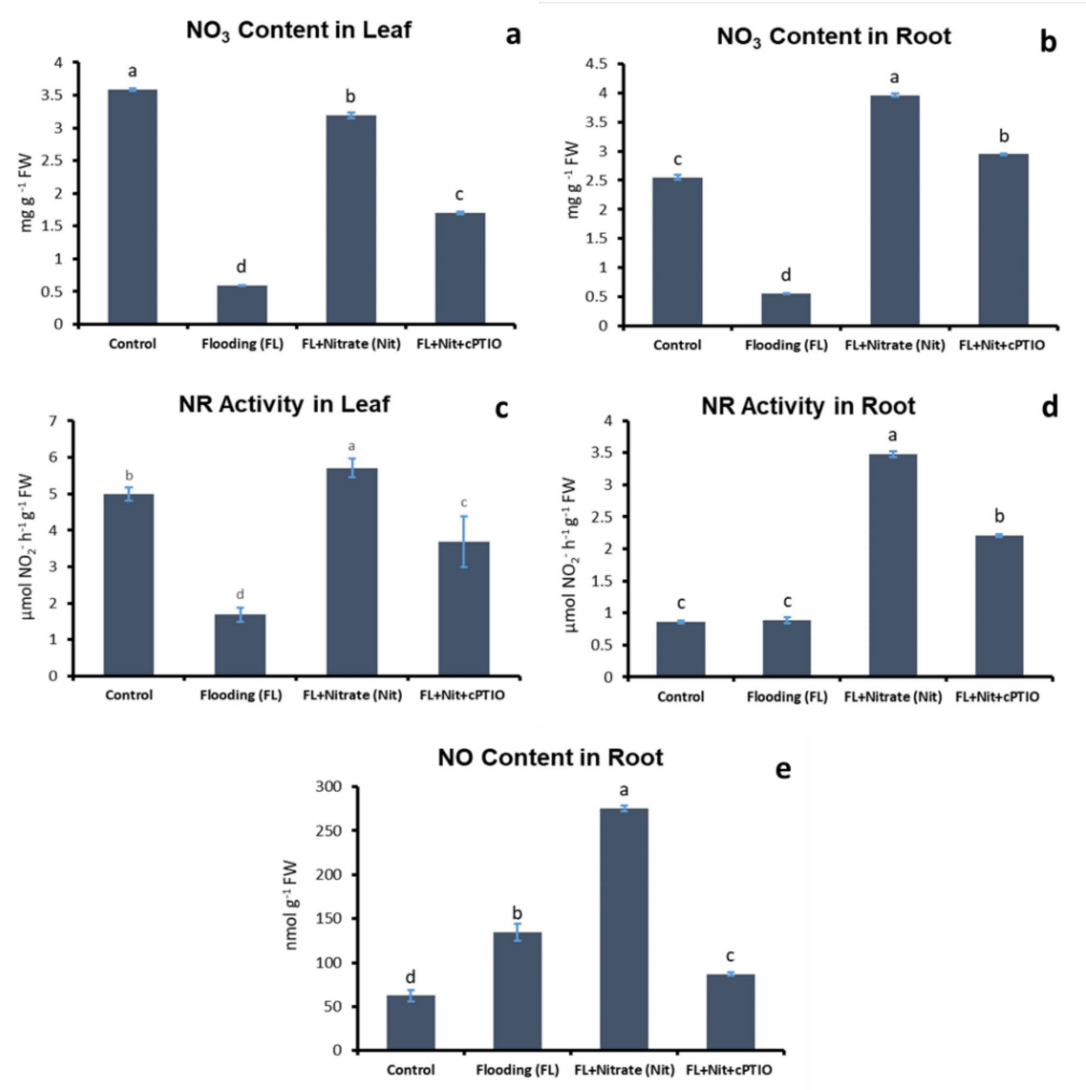
Nitrate content in leaf (a) and root (b), nitrate reductase activity in leaf (c) and root (d), and nitric oxide (NO) level (e) in root samples of control and waterlogged cucumber seedlings treated with nitrate (20 mM), and with nitrate plus cPTIO. Different letters on bars mark significant changes at 95% (*p* < 0.05) based on the Fisher’s least significant difference (LSD) test, used for comparison of the mean values. Labels and abbreviations are the same as in Fig. 3.

Waterlogging decreased the NO_3_^−^ concentration in the leaves, which was almost fully restored by nitrate application (Fig. 7a). When additional cPTIO was applied to waterlogged plants, the foliar NO_3_^−^ level declined partially to less than its half quantity of the untreated control (*p* < 0.05). The same pattern has been observed after NO_3_^−^ quantification in root samples (Fig. 7b). Waterlogging treatment reduced the nitrate content in leaves and roots significantly (*p* < 0.05) only when limited nitrate quantity was available in the flooding solution.

The nitrate reductase (NR) activity of leaves significantly decreased under WL stress (Fig. 7c). WL plus nitrate treatment increased it substantially (*p* < 0.05), even beyond its normal activity level. Additional cPTIO treatment decreased enzyme activity, but it did not sink down to the WL stressed level. Root NR activity followed a similar trend, with the difference that non-stressed activity was low, and it did not change significantly (*p* < 0.05) under WL stress (Fig. 7d). However, nitrate application elevated NR activity in roots too, which also declined when cPTIO was applied with nitrate.

Nitric oxide (NO) levels in roots increased in response to WL treatment and were further elevated by nitrate supplementation (Fig. 7e). Compared with nitrate treatment, scavenging of NO by cPTIO decreased the NO content significantly (*p* < 0.05) close to its basal level detected in control plants.

#### NO, H_2_O_2_ detection and protein nitration in roots

The visualization of endogenous NO and extracellular H_2_O_2_ in lateral cucumber roots was carried out using fluorescent probes and confocal laser scanning microscopy. According to the performed spectral unmixing (excitation at 488 nm, emission at 500–580 nm in 5 nm steps), strong green autofluorescence was observed in the cytoplasm of the outer layer of columella and lateral cap cells at the cell division zone, in the cytoplasm and cell walls of the epidermis at the transition and elongation (differentiation) zones of the lateral roots^25^ (Figure S1).

**Fig. 8:**
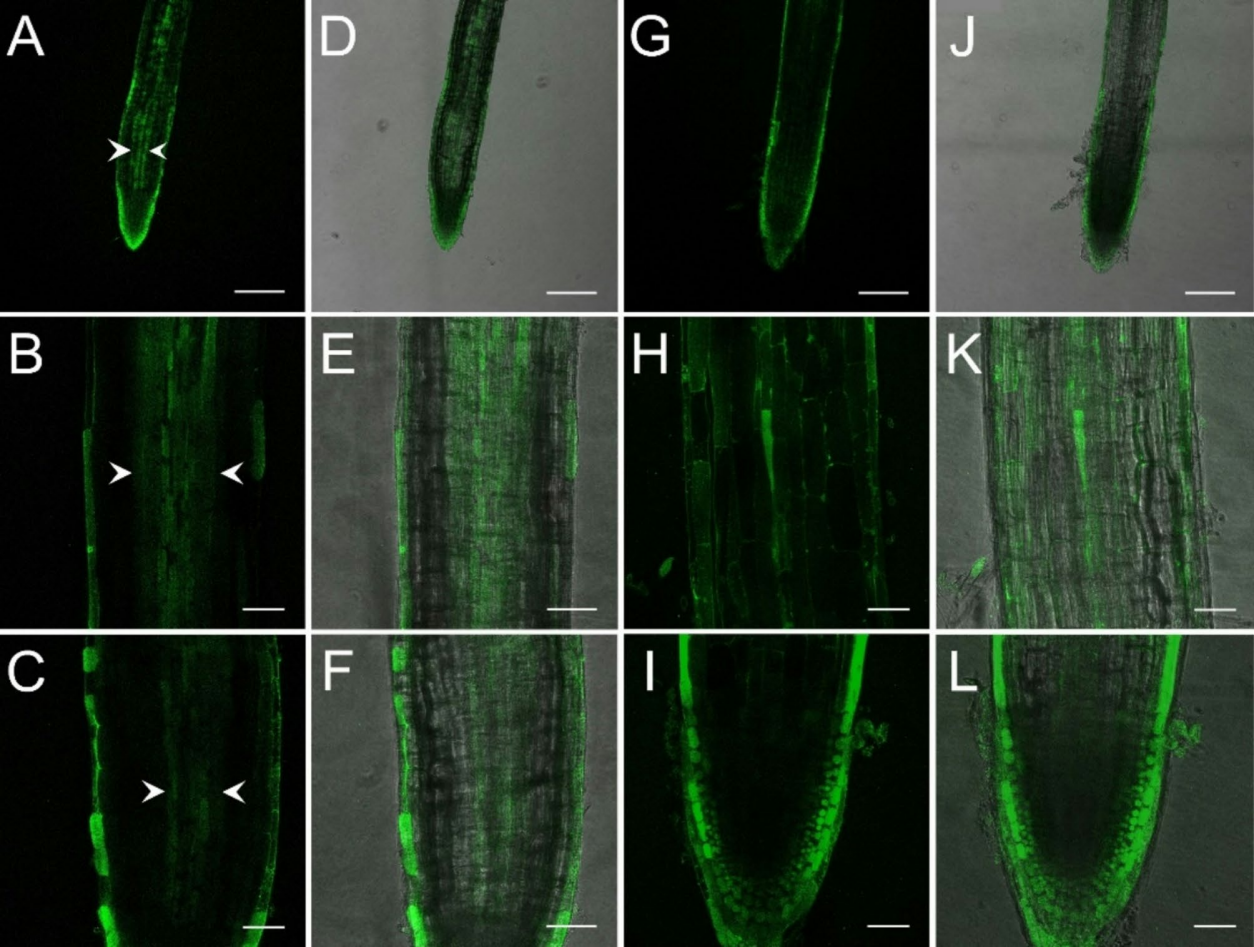
Localization of NO in WL/NO_3_^−^-supplemented (A-F), and SNAP-treated control (G-L) cucumber roots (representative images). A-C; G-I: fluorescent images; D-F; J-L: merged images. Arrowheads indicate the vascular cylinder. Bar = 250 μm (A, D, G, J); 50 μm (B, C, E, F, H, I, K, L).

In contrast, no green autofluorescence was present in the cortex or vascular cylinder (VC). Cucumber roots showed no NO accumulation in the cortex or VC of Control, WL only, or WL/NO_3_^−^/cPTIO. In contrast, NO was present along the cytoplasm and cell walls of the VC in 17% of the lateral roots of WL/NO_3_^−^ plants (Fig. 8).

**Fig. 9:**
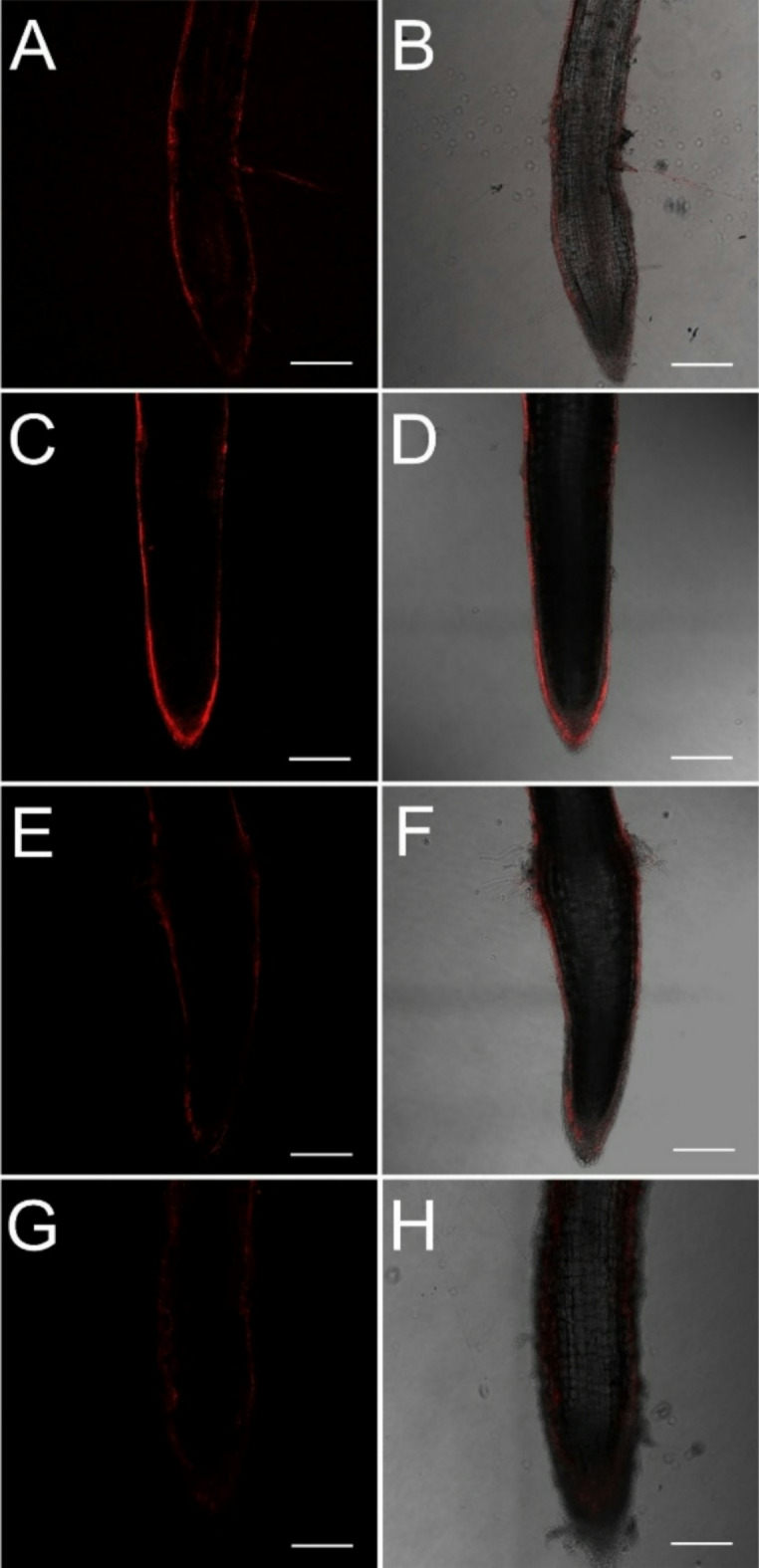
Localization of H_2_O_2_ in control (A, B), waterlogged (WL) (C, D), WL/NO_3_^−^-supplemented (E, F) and, WL/NO_3_^−^-supplemented and cPTIO-treated cucumber roots (G, H) (representative images). A, C, E, G: fluorescent images; B, D, F, H: merged images. Bar = 250 μm.

**Fig. 10:**
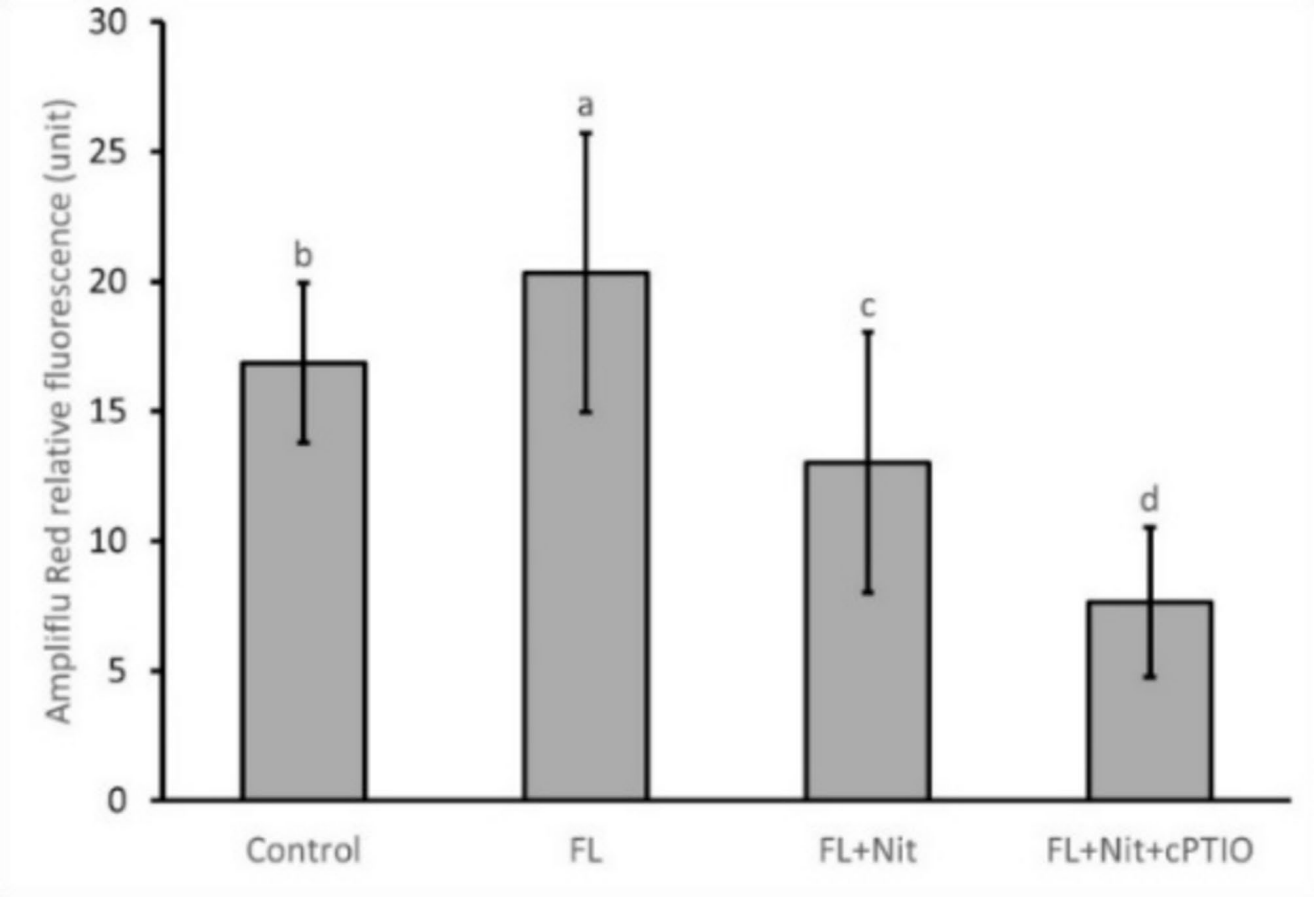
Relative fluorescence intensities were detected after Ampliflu Red labeling in the exodermis of cucumber lateral roots. Different letters on bars mark significant changes at 95% (*p* < 0.05) based on the Fisher’s least significant difference (LSD) test, used for comparison of the mean values. Labels and abbreviations are the same as in Fig. 3.

For H_2_O_2_ detection autofluorescence was also checked in the relevant spectral range. No red autofluorescence was detected in lateral roots when spectral unmixing was carried out in 5 nm steps with the excitation and emission wavelengths of 514 nm and 520–600, respectively. Compared to control roots, flooding enhanced while nitrate supplementation reduced the hydrogen peroxide content of the lateral roots by 21% and by 41%, respectively (Figs. 9 and 10).

**Fig. 11:**
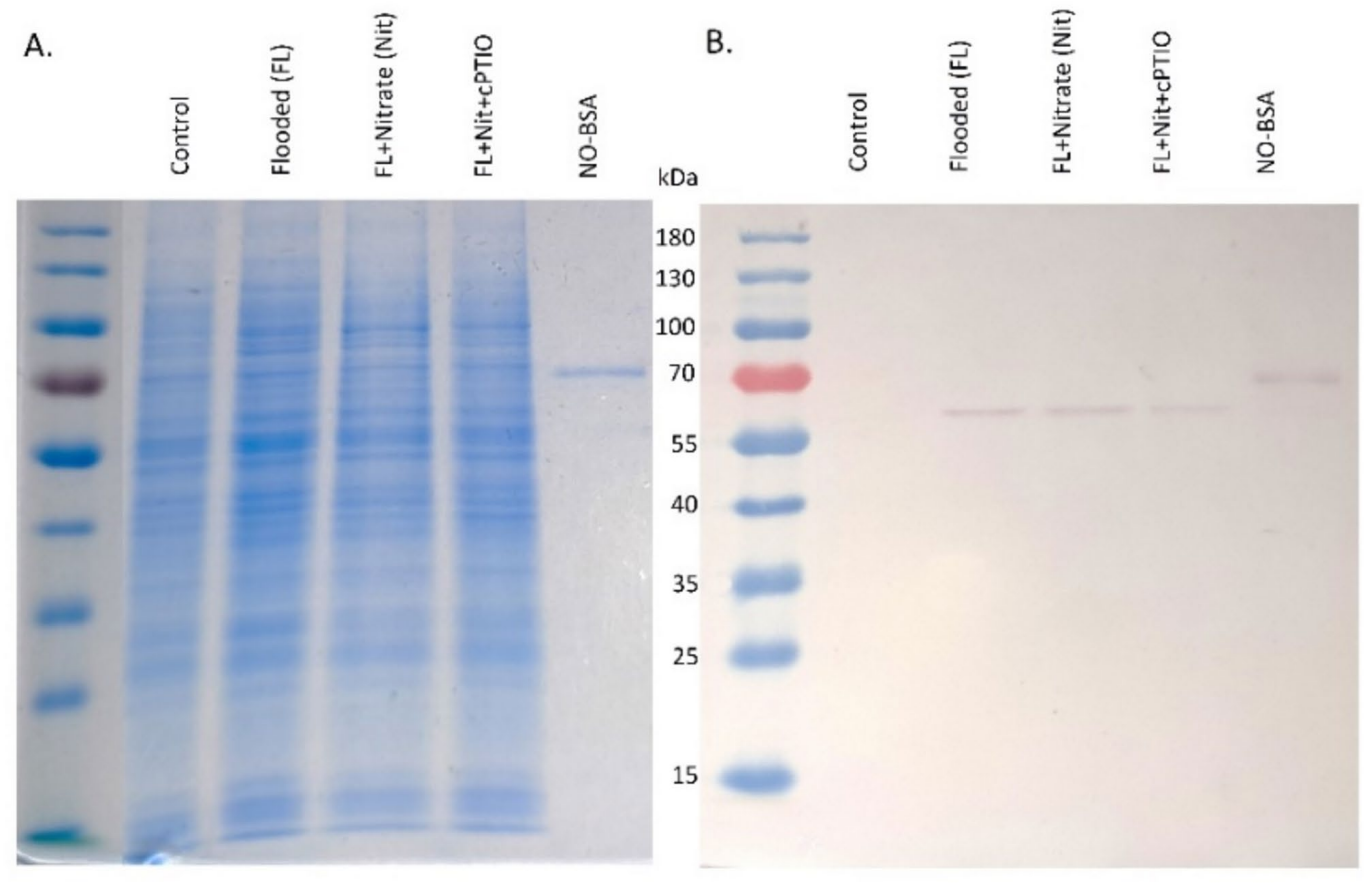
Western blot analysis of nitrated proteins isolated from cucumber roots. Coomassie Brilliant Blue stained protein gel (A), Immunoblot with antibody specific for nitrated proteins (B). Labels and abbreviations are the same as in Fig. 3. NO-BSA – nitrated BSA as positive control.

Protein nitration was detected by using an antibody against nitro-tyrosine in roots extracts. A single well-defined band indicated one major nitrated protein with a molecular weight of appr. 60 kDa in WL and WL/nitrate treated roots. Additional cPTIO treatment decreased the intensity of the signal. Specificity of immunoblot was validated with 0.5 µg nitrated-BSA (NO-BSA) (Fig. 11).

### Gene expression and signaling pathways

**Fig. 12:**
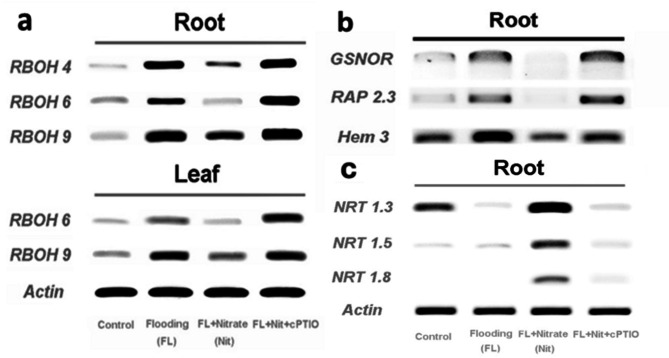
Representative semi-quantitative RT-PCR visualization of selected respiratory burst oxidase homolog (*RBOH*) and actin genes in root and leaf samples (a), followed by expression patterns of S-nitroso-glutathione reductase (*GSNOR*), *RAP2.3* transcription factor, and *Phytoglobin3* (*CsHem3*) genes (b) and that of nitrate transporter and actin genes in roots of differentially treated cucumber seedlings (c). Labels and abbreviations are the same as in Fig. 3.

Expression of nitrate transporters (NRTs) was studied on the genes reported by Migocka et al.^64^. Nitrate responsive *NRT1* genes that were expressed in root were amplified from cDNA samples prepared from root samples of seedlings from each treatment. Significantly higher expression of the selected NRT transcripts were observed after agarose gel electrophoresis in contrast to the stable expression of *Actin* gene transcripts (Fig. 12c).

Respiratory burst oxidase homolog (*RBOH*) genes expression was induced by WL stress in the roots (Fig. 12a). In all cases, the observed transcriptional induction was lower when nitrate was present in the flooding solution. This nitrate-associated-lower-expression of *RBOHs* was changed to their higher level of expression in WL treatments. The expression of *CsRBOH4* was not reliably detected in leaves, but in case of *CsRBOH6* and *CsRBOH9*, the same pattern of higher expression after flooding, lower expression after flooding plus nitrate, and again a higher expression after flooding plus nitrate plus cPTIO were observed in leaves of cucumber plants, relevant to the stable expression of the *Actin* gene (Fig. 12a).

**Fig. 13:**
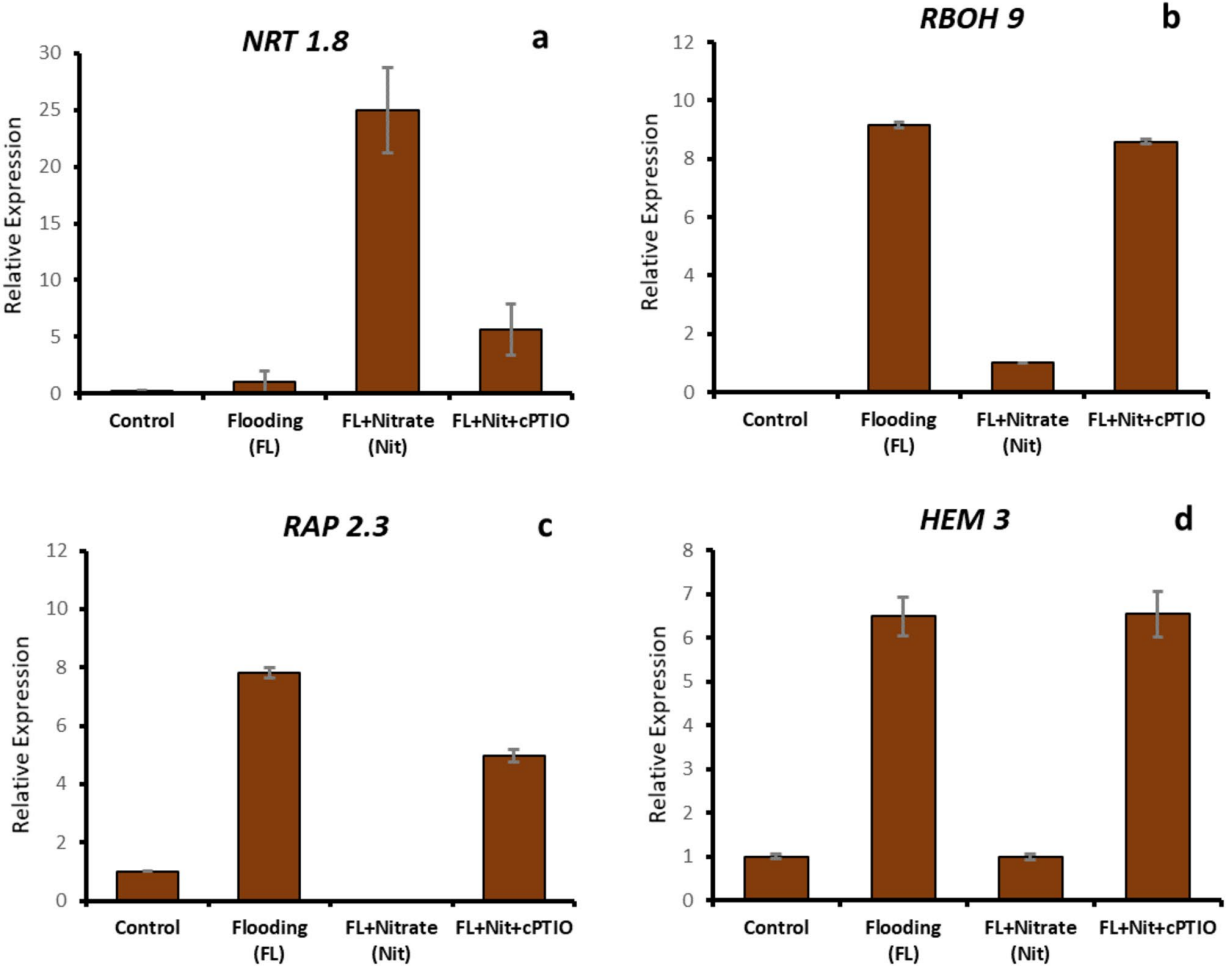
Validation of relative expression analysis of selected genes by qRT-PCR. Labels and abbreviations are the same as in Fig. 3.

Expression of the *CsRAP2.3* transcription factor gene was induced in roots by WL, which was reversed by nitrate supplementation and was induced again by cPTIO treatment (Fig. 12b). The same pattern repeated itself when the *Phytoglobin 3* (*CsHem3*) gene, coding for a putative plant hemoglobin, was RT-PCR amplified and visualized by electrophoresis (Fig. 8b). A candidate gene for S-nitroso-glutathione reductase (*CsGSNOR*) was induced by WL stress, repressed by WL/NO_3_^−^ treatment, and again strongly induced in WL/NO_3_^−^/cPTIO roots. RT-qPCR analysis was performed for some representative genes (*NRT1.8*, *RBOH9*, *RAP2.3* and *CsHem3*) to confirm the observed expression patterns in semi-quantitative RT-PCR (Fig. 13).

### Radar chart and correlation analyzes

Markedly, the nitrate level in roots and leaves showed a significant positive correlation (***p) with nitrate reductase activity (NR) in roots (*r* = 0.81) and leaves (*r* = 0.90). However, a negative correlation was confirmed between nitrate level in leaf with H_2_O_2_ (*r*= – 0.60), MDA (*r* = – 0.72), and ADH (*r* = – 0.57). The same negative correlation was also found between the nitrate reductase (NR) activity of leaf and the H_2_O_2_ (*r*= – 0.81), MDA (*r* = – 0.93), and ADH (*r* = – 0.83).

**Fig. 14:**
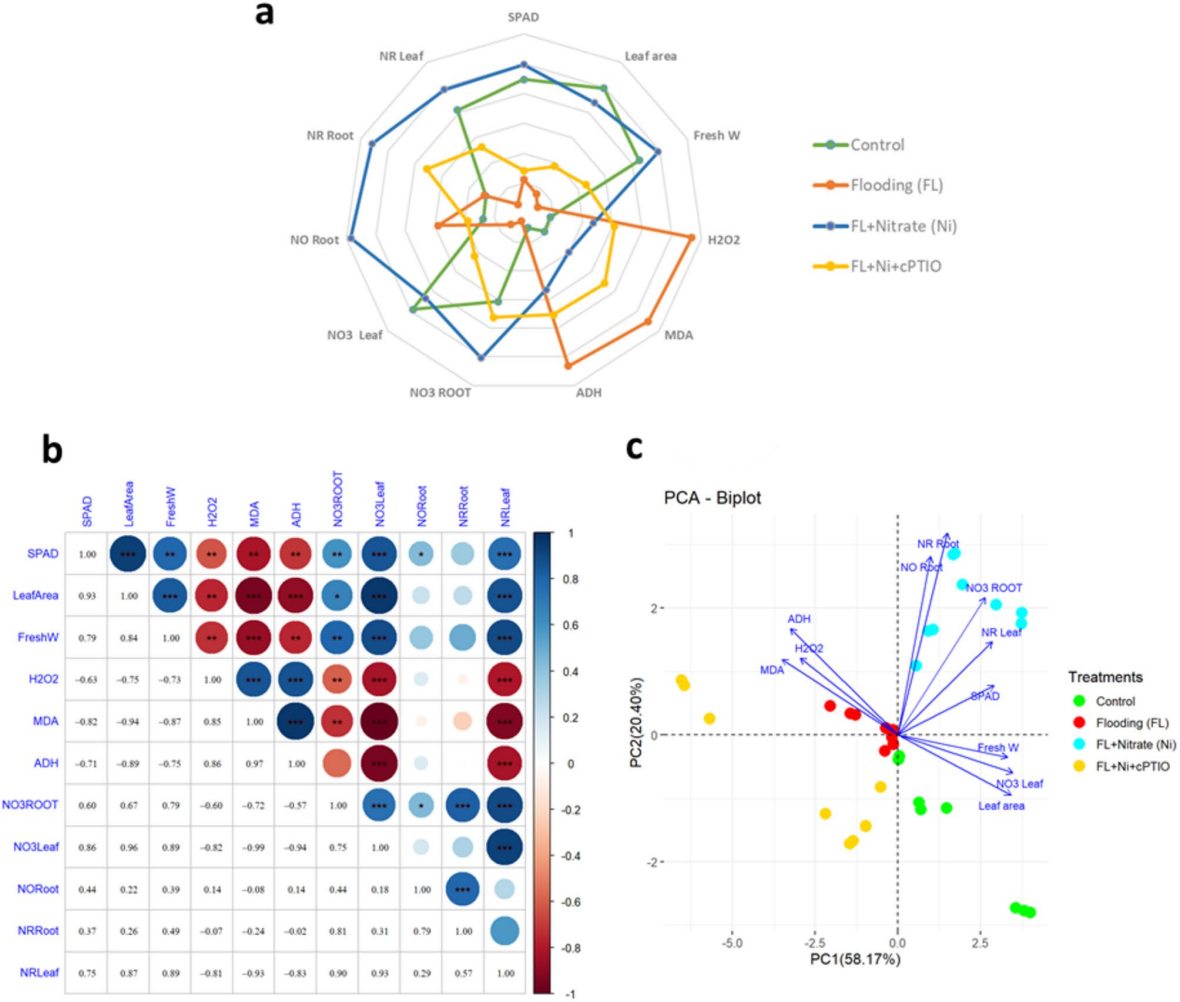
The radar chart **(a)**, the Pearson’s correlation analysis (**b**: *p, 0.05; **p, 0.01; ***p, 0.001), and the principal component (PCA) biplot **(c)** analyses of the applied treatments and their effects on the studied traits in cucumber seedlings. SPAD: SPAD value, Fresh W: fresh weight, H_2_O_2_: hydrogen peroxide concentration, MDA: malondialdehyde concentration, ADH: alcohol dehydrogenase activity, NO3 Root: nitrate content of the root, NO3 leaf: nitrate content of the leaf, NO Root: nitric oxide level in root, NR Root: nitrate reductase activity in root, NR Leaf: nitrate reductase activity in leaf. Labels and abbreviations of treatments are the same as in Fig. 3.

Accompanying the Pearson’s correlations test a principal component analysis (PCA) of the data was also performed and the first two PC were scattered on a biplot in order to expand our view on the variation distances of studied attributes (Fig. 14c). It turned out that the two first components (PC1 = 58.17% and PC2 = 20.40%) accounted for 78. 57% of the meaningful changes associated with original data. The comparison of the first and second components, revealed a bigger picture of overall positive correlation amongst the studied attributes except the hypoxia stress markers (H_2_O_2_, MDA, and ADH) which were negatively correlated with the rest of the studied traits. The first component can be considered as photosynthetic yield parameters and cell damage component, while the second component can be regarded as hypoxia stress indices, nitrogen associated attributes, and tolerance mechanism.

## Discussion

Consequences of nitrate supplementation in waterlogging (WL) stress were investigated in cucumber. Earlier work conducted on commercial F1 hybrids revealed that waterlogging caused surprisingly mild or no growth retardation when flooding was performed with high nutrition level media^6^. To follow up these studies we set out to explore which component of the media was effective in alleviating WL stress. For this purpose, a hybrid with moderate WL stress sensitivity (‘Joker’) was investigated in detail. In order to test its involvement, nitrate nutrition was supplied at normal and high concentrations under WL condition. In order to distinguish NO effects, cPTIO treatment was applied. As adventitious root (AR) induction was observed and implicated in mitigating root hypoxic stress^6^, AR formation was also followed.

WL stress in all treatments resulted in a sharp decline of dissolved oxygen level in the growth media (Fig. 2). Plants in ‘WL only’ treatment displayed stunted growth, decreased chlorophyll content (Figs. 3b, c and d and 4) and elevated malondialdehyde concentration (Fig. 6b) in leaf tissues, approving stress and lipid peroxidative damage. Accumulation of hydrogen peroxide (H_2_O_2_) increased in leaves of WL stressed plants, indicating redox imbalance (Fig. 6a). The above parameters were affected by WL, as was confirmed by correlation analyses (Fig. 14). As the plants had been subjected to WL already for 5 days at the time of the measurement, H_2_O_2_ production was not part of a temporary oxidative burst at the onset of WL stress^26^. Osmotic stress is known to promote ROS production^27^, thus it may be responsible for the oxidative response seen in the leaves. It can also be hypothesized that the observed H_2_O_2_ signal may have spread systemically from root to shoot, as proposed by Peláez-Vico and co-workers^28^. Indeed, *RBOH* genes (*CsRBOH4*,* 6* and *9)* were induced in WL treated roots, and coordinated upregulation of *CsRBOH6* and *9* was also found in leaves of the same plants. This situation raises the possibility of the existence of a persistent oxidative wave in WL. In WL stressed roots a shift to anaerobic metabolism was observed, indicated by increased alcohol dehydrogenase (ADH) activity (Fig. 3a). Above the level of the culture media ARs developed, which is a common response in WL stress. As ERF VII type transcription factors are putative regulators of hypoxia responses, they were identified in the cucumber genome. From this group a candidate gene was selected, complying with the N end rule. Transcription of the selected *CsRAP2.3* gene was indeed found upregulated in WL treated roots (Fig. 12b). In the roots of ‘WL only’ treated plants moderate accumulation of NO was observed by chemical analysis (Fig. 7e). In the same roots upregulated phytoglobin transcription indicated intensive operation of the Pgb/NO cycle, while induction of a *GSNOR* gene could also correspond to protection against the rising NO concentration (Fig. 12b). Nitrate induced, NO mediated post-translational protein modifications include Tyrosine nitration, which may be part of the nitro-oxidative damage in stresses and may modify target proteins in various ways. In general, it is considered damaging, as nitrated proteins tend to be degraded faster. On the protein level limited tyrosine nitration was detected in WL stressed roots (Fig. 11), what is ascribed to the elevated NO level. WL conditions strongly inhibited nitrate accumulation in leaves and roots (Fig. 7a and b), indicating decreased uptake and xylem transport, with low or downregulated nitrate transporters expression (Fig. 12c). Impaired nutrient uptake and efficiency is a well-known effect of WL stress^29^. Increased nitric oxide level in the flooded roots probably downregulated nitrate uptake and transport as was also described by Frungillo et al.^30^. As nitrate transport processes are highly energy dependent, acidosis and the overall energy crisis developing in hypoxic roots may cause their decline and downregulation. As NO exerts inhibitory effect on the mitochondrial electron transport chain^24^, it may generally contribute to the drop of energy supply and dependent functions, such as transport of minerals.

By elevated nitrate supply, shoot growth inhibition imposed by waterlogging was completely reversed. Growth parameters returned to normal, SPAD, H_2_O_2_ and MDA values improved in leaves (Figs. 3b, c and d and 4, and 6b). ADH activity decreased in the root (Fig. 3a). These changes indicated partial resolution of the energy crisis in the root and decreased stress level in leaves. Mitigating effect of nitrate over WL stress has been also observed in soybean^31^. Nitrate levels increased in leaves and roots of WL/NO_3_^−^ treated plants (Fig. 7a and b), along with induction of relevant transporter genes in the roots (Fig. 12c). Expression of nitrate transporters is highly regulated, with functions deeply connected to acclimation responses^32,33^. Nitrate dependent transcriptional upregulation of a set of genes, including nitrate transporters has been described as the primary nitrate response pathway (PNR). Nitrate has been implicated acting directly on transcriptional regulation in this process^2,34^. Under hypoxic conditions however, the operation of the transporters may face the problem of energy shortage. In the low oxygen level of hypoxic tissues various reductive pathways of NO formation may operate. These include NRases, xanthine oxidoreductase, alternative oxidases and members of the mitochondrial electron transport chain (mETC)^16^. From among these mETC probably dominates NO production in hypoxic plant tissues when nitrate is easily available^15^. According to a plausible scenario, WL/NO_3_^−^ plants’ energy supply could be improved by anaerobic respiration, which may alleviate energy deficiency and the need for fermentation in the flooded roots. In this process, nitrite may serve as electron acceptor, which is the most likely source of the increased NO content. NO was detected in WL/NO_3_^−^ treated roots both by chemical analysis and confocal fluorescent microscopy. According to microscopy, the NO produced was localized in the vascular cylinder of the roots. Although fluorescent microscopy detected the signal only in a subset of samples, we believe that the localization is correct, as the signal was specific and never occurred in other tissues or in different treatments. Resolution of the energy problem by anaerobic respiration in WL may not be universal, as the NO produced can have inhibitory effect on nitrate reductase and cytochrome c oxidase activities, what normally restricts its extensive activity^20,24^. It seems that this constraint is relaxed in the cucumber hybrid investigated, allowing the observed increase of NO without severely limiting mitochondrial functions or nitrate assimilation. The molecular details of this intriguing behavior are not yet known but certainly merits further investigations. In this situation the growth promoting effects of NO on development and metabolism may prevail, overcome inhibitory effects and result in stress alleviation. NO has well-characterized effects on root development, including root porosity, aerenchyma, lateral and adventitious root (AR) formation^36–38^. From among those, ARs were investigated here, which were induced by WL (Fig. 5), as was already shown in our earlier work^6^. Here we demonstrated that this induction was further enhanced by nitrate supplementation, and partly reverted by NO scavenging. Therefore, our data confirm earlier observations, approving that AR induction is promoted by nitric oxide. AR formation can improve internal oxygen supply of root tissues and thus elevate their aerobic respiration activity, decreasing the need for fermentation. The partial resolution of oxidative stress in the shoot in WL/NO_3_^−^ treatment also indicates improved root function. A healthier energy balance probably supported mineral transport, as signs of stress disappeared along with growth retardation in the shoot of WL/NO_3_^−^ treated plants. ARs have probably contributed to the better root function in our nitrate treated WL stressed cucumber plants. However, although partial hypoxia was still present in WL/NO_3_^−^ treated roots (according to ADH activity), their growth was fully ameliorated. Therefore, it is evident that some process other than aerobic respiration also improved the energy status of the root. This process was most likely anaerobic respiration, also accountable for the observed rise of NO levels in WL/NO_3_^−^ treated roots. Nitrate dependent improvement of root hydraulic functions has been already described^39–41^. In WL/NO_3_^−^ treated roots downregulated expression of *CsRAP2_3*, a proposed ERFVII type regulator, was found compared to “WL only” treatment (Fig. 12b). Potential targets of this transcription factor, alcohol dehydrogenase activity and a phytoglobin gene (*CsHem3*) were also repressed in roots under WL/NO_3_^−^ treatment (Figs. 3 and 12b). Partially improved oxygenation may explain downregulation of *CsRAP2_3* in this treatment. High NO level can also trigger repression of ERFVII transcription factors^42^. Moreover, energy status may also affect hypoxia regulated gene expression through the LACS/ACBP regulatory pathway, which can directly respond to changing ATP levels^43^. Although the LACS/ACBP and the N-end rule regulatory pathways operate at the post-translational level, we hypothesize that in WL/NO_3_^−^ treated roots transcription of relevant TFs, such as *CsRAP2_3*, may have been affected as well. Nitrate provision restricted WL induced hydrogen peroxide and MDA levels in the roots and leaves substantially (Figs. 6 and 9). Less ROS production and lipid peroxidation in the shoot reflect a resolution of redox balance there and could result from improved root functions. The high NO content in roots may downregulate ROS production, leading to decreased oxidative stress and signaling towards the shoot (see below).

In order to dissect nitrate induced alleviation of WL stress cPTIO, a known NO scavenger was applied. This compound was delivered to roots and leaves combined, to ensure withholding all NO dependent processes at both locations. cPTIO blocked the beneficial effect of nitrate on shoot growth (Fig. 3c and d), indicating that NO is essential for restoring leaf growth by nitrate in WL. The mitigative effect of NO in hypoxic stress has been already documented, with several potential mechanisms put forward, also in cucumber^16,33,44^. In our system cPTIO could remove most of the NO produced in the root (Fig. 7e). GSNOR activity is regarded as regulator of NO homeostasis, depleting excess NO through GSNO conversion^23^. The putative *GSNOR* gene identified in this study displayed characteristic transcript profile changes through the treatments. While it was downregulated in WL/NO_3_^−^ plants, it was upregulated by WL/NO_3_^−^/cPTIO treatment (Fig. 12b). This pattern suggests a transcriptional regulatory link with *CsRAP2_3*, rather than being regulated by NO level per se. However, these data leave open the possibility for regulation at the enzyme activity level. Interestingly, WL/NO_3_^−^/cPTIO treatment inhibited transcriptional induction of the nitrate transporter genes in roots, compared to WL/NO_3_^−^ plants (Fig. 12c). Therefore, it became evident that NO played a decisive role in induction of nitrate transporters’ gene expression in WL/NO_3_^−^ treated cucumber roots. The central role of NO in the primary nitrate response in normoxia has also emerged recently^45^. Our results confirm a positive regulatory role of NO on root *NRT1* genes expression in the context of waterlogging stress. These genes are likely member of the low affinity transport system (LATS) for nitrate transport in cucumber. Because of the metabolic connections the effect of NO on nitrogen acquisition has been studied in other species as well (reviewed by^46^). NO induced expression of nitrate transporters and resulting increase of nitrogen uptake capacity was found in tobacco^47^, rice^48^, common beech^49^ and Scots pine^50^. However, in some other studies NO was found suppressing nitrate transporters, leading to a negative feed-back loop on nitrate assimilation^30^. Surprisingly, repressed *NRT1* gene expression in WL/NO_3_^−^/cPTIO plants did not lead to a return to the basal foliar nitrate levels (Fig. 7). According to RT-qPCR data, expression of *NRT1* genes was not completely blocked (Fig. 13). Thus, low mRNA level potentially could still support sufficient protein synthesis to maintain transporter function. It is more likely though, that decreased uptake/transport and less intensive incorporation of nitrogen (e.g. low NRase activity, see Fig. 7d) may slow down turnover of the nitrate pool in a way that result in close to normal nitrate concentrations in WL/nitrate/cPTIO treated plants. Besides nitrate transport, probably several other NO dependent steps are also involved in leaf growth promotion^23^. Earlier studies linked nitrate regulated growth promotion to hormonal effects^51^. NO is well known to interact with a range of plant hormones thereby shaping responses in waterlogging^11,23^. As some examples, metabolism and signaling of ethylene, abscisic acid and salicylic acid are known to be influenced by NO, what may have consequences in WL situation. It is therefore likely that downstream effects of NO also involve interferences with hormonal pathways in WL/NO_3_^−^ plants. These interactions may occur in root, shoot or at both places, impacting on growth^52,53^. Details of these molecular events need elucidation through further studies. NO content of the root may also influence redox equilibrium of the whole plant. *RBOH* genes got activated by WL and inactivated by WL/NO_3_^−^ treatments (Fig. 12a). In WL stressed roots increased abundance of extracellular H_2_O_2_ was detected in the exodermis by fluorescent microscopy, which signal was decreased by WL/NO_3_^−^ and WL/NO_3_^−^/cPTIO treatments (Figs. 9 and 10). This strengthens the assumption that a root derived extracellular oxidative signal may reach the shoot and at least contributes to signaling for stress tolerance. Apparently, NO may dampen this presumed signal by inhibiting *RBOH* gene expression as was described by Sasidharan et al.^54^, for RBOH activity. Direct oxidative effects may impact on shoot redox status. Changing oxidative balance of leaves raises the possibility of redox regulation of growth^55^. However, H_2_O_2_ content of leaves did not correspond well with growth, making this a less likely alternative. As we could not measure NO level in leaves, it is difficult to reconcile NOs direct influence on foliar redox balance, which is probably exerted in a complex way even locally^23^.

**Fig. 15:**
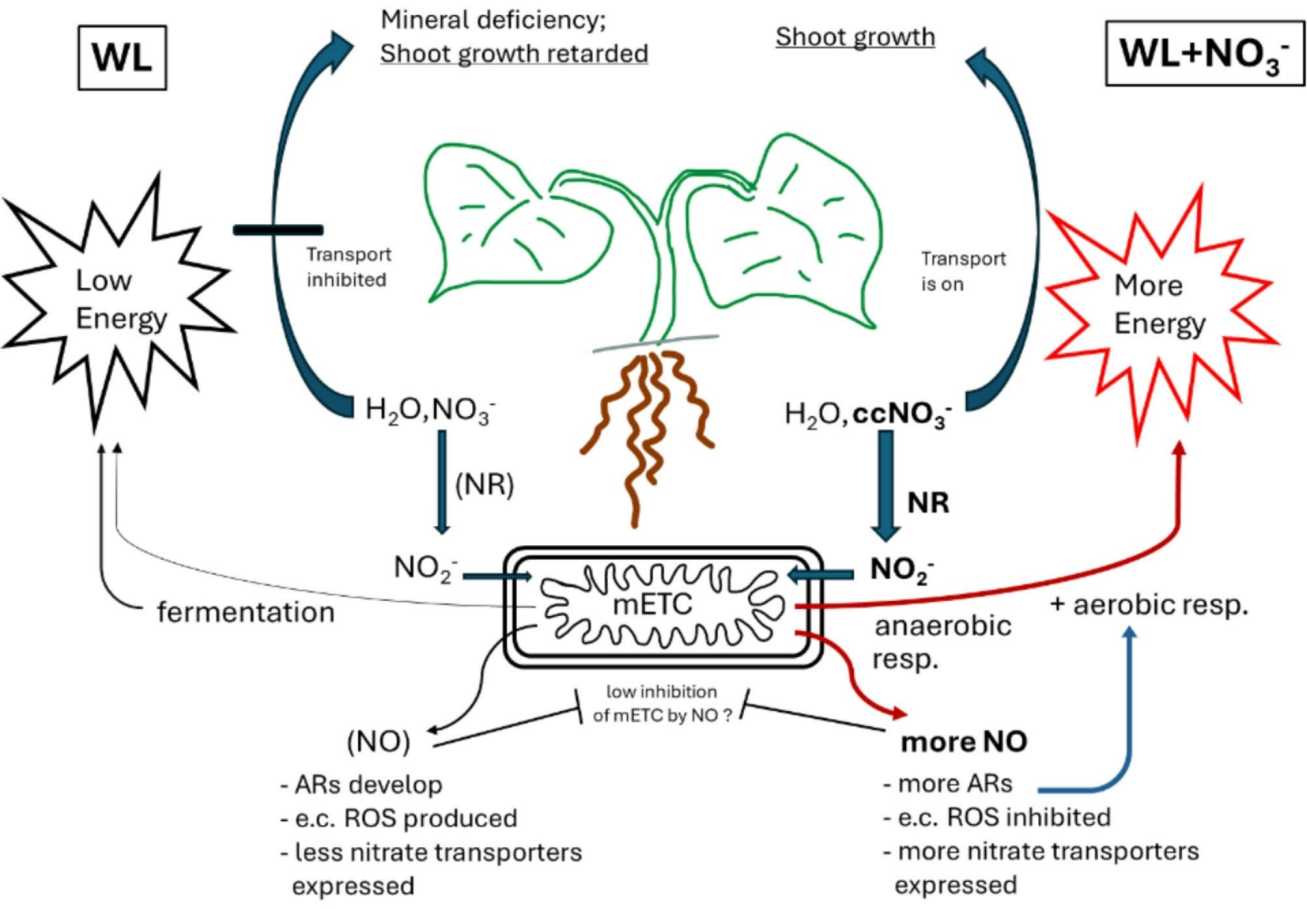
Events supported by the present study, related to nitric oxide-based regulation in cucumber hybrids in WL stress and its mitigation by nitrate.

Conclusions and perspective: In summary, our research revealed nitrate induced, NO mediated alleviation of growth retardation in WL stressed cucumber. WL resulted in a shift towards fermentative metabolism in the hypoxic roots and caused stunted shoot growth. This was paralleled by signs of oxidative stress in roots and shoot. A set of *RBOH* genes was induced at both locations. H_2_O_2_ accumulated in the extracellular space of the root’s exodermal tissue, and in the leaves. The contribution of a systemic oxidative signal was implicated as causing oxidative effects. Nitrate (20 mM) supplemented flooding solution mitigated WL stress, based on growth parameters and oxidative markers. Nitrate treatment accelerated AR formation, which probably contributed to internal aeration of the root. Although substantial ADH activity indicated that these root tissues were still partially hypoxic, they supported intensive growth. Anaerobic respiration with nitrite as electron acceptor was proposed as a mechanism to improve energy balance and functions of the flooded roots. This process could backup energy dependent root functions, such as mineral transport. This type of anerobic respiration may yield NO, which was found accumulating in the roots, localized to the vascular cylinder by fluorescent microscopy. It was revealed for the first time that the NO produced in WL can mediate transcriptional upregulation of nitrate transporter (*NRT1)* genes, likely expanding transport capacity from roots to shoot. With the appropriate energy supply available and transporters expressed, nitrate transport could be facilitated toward foliar tissues (Fig. 15).

Restoring foliar nitrate level and resolving the energy crisis in WL stressed roots probably played major roles in facilitating growth. Further growth promoting effects of the NO produced may be directly and indirectly reducing the oxidative stress arising in flooded roots and shoot. Development of ARs is also enhanced by NO, which probably facilitated increased aerobic respiration in root tissues. These NO mediated effects most likely contributed to the mitigating influence of nitrate in WL stress.

The results presented here need to be complemented by morpho-physiological studies to reveal any changes in the structure of tissues involved. Hormonal aspects should also be explored, that are probably highly relevant and could be linked to the NO effects investigated here.

## Methods

### Plant materials and growth conditions

This study utilized an open field hybrid F1 cultivar (‘Joker’) of cucumber in a semi-hydroponic growth system. Seeds were procured from ZKI Ltd., Hungary, and immersed in distilled water for 24 h at 25 °C before being cultivated in soilless media. Seeds were sown in rockwool cubes that were placed in the center of 9 × 6.5 × 6.5 cm pots containing 50 g of perlite in quadruplicate. These pots were then transferred to a phytotron (FitoClima 600, Portugal) for germination and grow under controlled conditions: 27 ± 1 °C during the day and 20 ± 1 °C at night, with a 16-hour photoperiod and a photosynthetic photon flux density (PPFD) of 150 µmol m^–2^ s^–1^, which was provided by cool-white fluorescent lamps. The relative humidity in the growth chamber was 75–80%. Hoagland fertigation solution was applied every day (50 ml per plant) after the emergence of the first true leaf (8 days after seed sowing) until the end of the experiment. The content and composition of the applied half-strength Hoagland solution is shown in Table S1.

### Stress conditions and treatments

A schematic illustration of the experimental design and treatments is shown in Fig. 1. Waterlogging was applied at day 16 after germination by inserting the pots in polyethylene containers and submerging them appr. 2 cm below the liquid surface in different flooding solutions (deionized water as control, 20 mM KNO_3_ as nitrate treatment, and 20 mM KNO_3_ + 10 µM cPTIO (C_14_H_16_KN_2_O_4_) as NO scavenger treatment). The waterlogging stress continued for 5 days. During this time the plants that were flooded with 20 mM KNO_3_ + 10 µM cPTIO solution also received 10 µM cPTIO via foliar spray until leaf drop every day. Fertigation was applied every other day as 50 ml half strength Hoagland solution per plant until the end of the experiment. The O_2_ concentration (DOI) of the flooding solutions was regularly recorded using an oxygen meter device (Voltcraft DO-101, Conrad Electric SE, Germany). Evaporation of the flooding media was minimized by covering the liquid surface with polystyrene spheres (2–6 mm in diameter). At day 21 (corresponding to 5 days post flooding) AR numbers were counted above the surface of the growth substrate, with the help of a magnifying glass. At least three independent plants were used for each analysis requiring fresh tissues. After recording the chlorophyll content (SPAD 502; Minolta Camera, Osaka, Japan), fresh weight was measured on a laboratory scale. H_2_O_2_ and NO levels were determined from leaves and roots, respectively. The remaining leaf and root samples were instantly frozen in liquid N_2_ and stored at – 80 °C for further analyses and RNA extraction. Leaves of some plants were photographed and total leaf area per plant was calculated with the ImageJ software (version 1.53t, Image Processing and Analysis in Java, Bethesda, MA, USA).

### Determination of hydrogen peroxide and lipid peroxidation

The H_2_O_2_ content in the leaves was quantified as follows. A colorimetric reaction method was used as previously described by Mosa et al.^56^. Grounded leaves (0.2 g) were mixed with 4 ml of 0.1% TCA (w/v), and the homogenate was centrifuged at 13,000 rpm for 15 min. Supernatant (0.5 ml ) was mixed with 0.5 ml of potassium phosphate buffer (10 mM, pH 7) and 1 ml of 1 M potassium iodide (KI) before checking their absorbance at 390 nm by using a spectrophotometer. The H_2_O_2_ content was determined in nmol g^–1^ FW using a calibration curve of H_2_O_2_ standard solutions.

The content of MDA in leaves was quantified using the TBA (thiobarbituric acid) method of Hodges et al.^57^ with slight modifications. In summary, leaf samples were extracted with 0.1% (w/v) TCA. The reaction with 0.5% (w/v) TBA in 20% (w/v) TCA was carried out at 100 °C for 30 min. Subsequent to cooling the samples on ice, the absorbance was measured at 532 nm, 600 nm, and 440 nm. The results were calculated by considering the absorption coefficient of 1.55 mM^–1^ cm^–1^ and finally presented as nmol MDA per g FW.

### Determination of NO and nitrate content

Nitric oxide (NO) was quantified by determining nitrite content according to the method of Mostofa et al.^58^. Root tissues (1 g) were grinded in mortars and pestles with 2 ml of cold acetic acid buffer (50 mM, pH 3.6) containing 4% zinc diacetate. The homogenates were subjected for centrifugation at 4 °C and 13,000 rpm for 15 min. The supernatant was treated with charcoal. Supernatant (1 ml) was mixed with 1 ml of the Griess reagent (1% sulphanilamide and 0.1% N-1-napthylethylenediamine dihydrochloride) and incubated at room temperature for 30 min in dark. The absorbance of the incubated reaction solution was recorded at 540 nm and the NO content was determined using a calibration curve established with different concentrations of sodium nitrite (NaNO_2_) as standard.

Nitrate (NO_3_^−^) concentration in the plant tissues was determined following the ISO standard (ISO 6635)^59^ using the salicylic-sulfuric acid method, which was previously described^60^. In summary, leaf and root samples (0.3 g) were grounded and placed in a flask with 4 ml of deionized water. The mixture was heated in a hot water bath (98 °C) for 15 min, diluted with 10.6 ml of deionized water, 0.2 ml of Carrez solution I (22% Zn acetate), and 0.2 ml of Carrez solution II (15% K_4_[Fe(CN)_6_] × 3H_2_O) and allowed to precipitate the interfering colloids and proteins. The 15-ml reaction solution was then vortexed and filtered. The filtrate (5 ml) was mixed with 0.5% Na-salicylate (1 ml) and heated in glass Petri dishes to dryness in an oven at 100 °C. Subsequently, the dried cooled residue was dissolved in 1 ml of sulfuric acid and then diluted with 3 ml of deionized water. The diluted acidic solution was then mixed with 7 ml of 10 M NaOH in flasks, which were then filled with 50 ml of deionized water. The resulting yellow solution, formed by the nitration of salicylic acid in strongly acidic environment was measured using a spectrophotometer at 410 nm. The NO_3_^−^content was quantified based on a calibration curve of sodium nitrate standard solutions.

### Nitrate reductase enzyme activity (NR)

NR activity was measured using the method outlined by Kim and Seo^61^. Leaf samples (0.2 g) were immersed in 750 µl of extraction buffer that consisted of 250 mM Tris-HCl (pH 8.0), ethylenediaminetetraacetic acid 1 mM), Na_2_MoO_4_ (1 µM), 5 µM FAD, dithiothreitol (3 mM), 1% BSA, 2-mercaptoethanol (12 mM), and phenylmethylsulfonyl fluoride (250 µM). After homogenization, the samples were centrifuged for 20 min (13,000 rpm, 4 °C), when the supernatant was collected. The enzymatic reaction was started by combining 150 µl of crude enzyme extract with 850 µl of prepared reaction buffer (NaNO_3_ (40 mM), Na_2_HPO_4_ (80 mM, NaH_2_PO_4_ (20 mM), NADH (0.2 mM)), to be proceeded at room temperature for 2 h. Afterwards, 200 µl of 1% sulphanilamide and 200 µl of 0.05% N-(1-naphthyl) ethylene diamine hydrochloride were gently introduced to the reaction mixture and kept at room temperature for 15 min. Enzyme activity rate was quantified at 540 nm using a spectrophotometer and is expressed as µM NO_2_ g^–1^ FW h^–1^.

### Alcohol dehydrogenase enzyme activity

The assessment of alcohol dehydrogenase (ADH) enzyme activity involved spectrophotometric measurements, wherein NADH oxidation at 340 nm was monitored following the procedure outlined by Kang et al.^62^. Root tissue samples (100 mg) were homogenized in a mortar with the addition of 500 µl of 50 mM Tris–HCl extraction buffer (pH 6.8), comprising MgCl_2_ (5 mM), mercaptoethanol (5 mM), glycerol (15% v/v), EDTA (1 mM), and PMSF (0.1 mM). To assay the ADH activity, a reaction mixture including 50 mM TES (pH 7.5), 0.17 mM NADH, 0.2% acetaldehyde (v/v), was reacted with 100 µl of enzyme-containing extract. The enzyme activity was quantified based on the molar extinction coefficient for NADH (6.22 mM^–1^ cm^–1^) and expressed as µM NADH min^–1^ g FW^–1^.

### NO and H_2_O_2_ detection by confocal laser scanning microscopy

At the end of the treatment period 6 mm long terminal ends of lateral roots were isolated from control and treated plants. The roots were incubated in Tris-HCl buffer (10 mM, pH 7.4) containing 10 µM DAF-FM DA (Merck Life Science Kft., Budapest, Hungary) for 60 min or 10 µM Ampliflu RED and 0.2 U/ml horseradish peroxidase for 30 min to detect NO and H_2_O_2_, respectively, at 25 °C in the dark. After incubation, samples were washed 3 times for 10 min each in Tris-HCl buffer. Spectral unmixing was performed at excitations at 488–514 nm, and emissions at 490–590 nm or 520–600 nm, in 5 nm steps on unlabeled cucumber roots (*n* = 5 per genotype and treatment) to detect autofluorescence overlapping with the excitation range of DAF-FM DA or Ampliflu RED, respectively. As controls of NO production, roots were pre-incubated before adding the fluorescent probe with 100 µM SNAP as NO donor, 100 µM cPTIO as NO scavenger, and 5 mM L-NAME as a competitive inhibitor of animal NOs. Similarly, before H_2_O_2_ detection with Ampliflu RED, roots were pre-treated either with 10 mM H_2_O_2_ (positive control), 200 U/ml catalase + 1 mM Ascorbic acid (scavenger), 10 µM DPI (NADPH-oxidase inhibitor) or 10 mM Na-azide (peroxidase inhibitor). Each compound was dissolved in 10 mM TRIS-HCl (pH 7.4). After staining and washing, roots were immediately mounted on microscope slides and examined with an SP8 confocal laser scanning microscope (Leica Microsystems GmbH, Wetzlar, Germany) using excitation at 488 nm and detection wavelengths 500–580 nm for DAF-FM DA and 561 nm/570–650 nm for Ampliflu RED. The laser intensity, and HyD detector settings (spectra, gain) were held constant at each magnification during the experiments to obtain comparable data. The fluorescence intensities of the images were analyzed using the LAS X software (Leica Microsystems GmbH, Wetzlar, Germany). Relative fluorescence intensity in the elongation zone was measured on 27 micrographs per genotype and treatment, using 6 independent regions of interest along the exodermis per micrograph. The fluorescence intensity data were normalized against the background fluorescence. The whole experiment was repeated twice with samplings in 3 biological and min 3 technical repetitions per treatment each time.

### Extraction of total RNA, cDNA synthesis, RT-PCR and qRT‒PCR

Leaf and root samples (0.5 g) were homogenized in liquid nitrogen using sterile mortar and pestle to obtain total RNA through a CTAB-based protocol^63^. The integrity of the secured RNA was confirmed on an EcoSafe-stained 1% agarose gel. RNA quantification took place using a NanoDrop 1000 spectrophotometer at 260 nm. All samples were subjected to DNase I treatment (Thermo Scientific) to remove genomic DNA, and the concentrations of RNA were adjusted to 5 µg per 50 µl. The integrity of the DNase-treated RNA was once more assured by gel electrophoresis prior to reverse transcription. First-strand cDNAs were produced according to the supplied protocol for the Maxima H Minus Reverse Transcriptase kit (Thermo Scientific) with oligo(dT)_20_ primers. List of the selected genes from cucumber genome and primer sequences are presented in Table S2. *CsNRT* primers were from Migocka et al.^64^. Amplification of the gene fragments were tested by RT-PCR using GO Taq G2 DNA polymerase (Promega, USA) in a Master Cycler instrument (Eppendorf AG, Hamburg, Germany) with the following cycling parameters: 3 min at 95 °C, 28 cycles of 15 s at 95 °C, 30 s at 56–60 °C, 30 s at 72 °C, and a final extension for 7 min at 72 °C. Amplified fragments from genomic DNA and cDNAs were visualized on a 1.2% (w/v) ethidium bromide-stained agarose gel in 1×TBE buffer.

qRT- PCR was performed in a CFX96 Real-Time PCR System (Bio-Rad, USA) using the SsoAdvanced Universal Inhibitor-Tolerant SYBR^®^ Green Supermix (Bio-Rad, USA). For relative gene expression analysis, the *Actin* gene (with its stable expression confirmed in our earlier studies, Oszlányi et al.^65^, and Szegő et al.^66^, was used as internal standard gene. PCR amplification was begun with polymerase activation and DNA denaturation at 95 °C for 30 s, followed by 40 cycles of denaturation at 95 °C for 10 s, and annealing and extension at 60 °C for 30 s. PCR product specificity was assessed by a terminal melting curve analysis (65–95 °C). Fold changes in gene expression levels were estimated by the built-in 2^−∆∆Ct^ method of the Bio-Rad CFX Maestro software.

### Western blot analysis of nitrated proteins

Root samples were homogenized in a fourfold volume of extraction buffer (50 mM Tris-HCl, pH 7.8, 1 mM EDTA, 1% (v/v) protease inhibitor cocktail, 0.1% triton X-100, and 10% (v/v) glycerol) according to Kolbert et al.^67^. Protein solutions were concentrated by Pierce 9 K MWCO (Thermo Scientific). One hundred micrograms of denatured protein extract were subjected to two parallel SDS-PAGE on 10% acrylamide gels. The proteins separation took place in a BioRad Mini-PROTEAN 3 system (BioRad, Hercules, USA) according to Laemmli^68^. Separated proteins in of the gels was visualized with GelCode Blue Safe Protein Stain (Thermo Fisher Scientific) and the other gel was blotted onto a nitrocellulose membrane (BioRad) according to the manufacturer’s instructions. The applied membrane was probed with rabbit polyclonal antibody against 3-nitrotyrosine (Sigma-Aldrich, USA) diluted 1:2000. Subsequently, the membrane was coupled with alkaline phosphatase-conjugated secondary antibody (AffiniPure Goat Anti Rabbit IgG, Jackson I.R. Laboratories Inc., USA) at 1:5000 dilution rate. Bands were visualized after 5-bromo-4-chloro-3-indolyl phosphate (BCIP)/ nitro blue tetrazolium (NBT) reaction. Specificity of the immunoblot was validated by nitrated bovine serum albumin (BSA). A 5 mg ml^–1^ BSA solution was made, and the pH was adjusted to 3.5 with acetic acid. Sodium nitrite (200 mM) was added at a final concentration of 1 mM and the solution was incubated rotating at 37 °C for 24 h. After that, the nitrated BSA solutions were dialyzed overnight to PBS and used as a positive control. The Western blot was applied on two protein extracts from two different biological repeats of the experiment, which yielded similar results.

### Statistical analysis

The experimental design was a completely randomized design with at least three biological replications. A one-way ANOVA was conducted to test significant differences among treatments. When a significant effect was detected, Fisher’s Least Significant Difference (LSD) test was used at 95% (*p* ≤ 0.05) level of probability for pairwise comparisons of the mean values using R software (v 4.4.0) on RStudio (v 2024.04.2). The Pearson Correlation Analysis was carried out by corrplot and Hmisc R packages. Spider (Radar) plot was created using fmsb package^69^ in R studio software and recreated in Excel 2024. The principal component analysis ((PCA)-Biplot) was constructed using the FactoMineR^70^, ggplot2^71^, and factoextra^72^ R studio packages. For the spider plots, the values were transferred to their corresponding Z-scores.

## Electronic supplementary material

Below is the link to the electronic supplementary material.

Supplementary Material 1

## Data Availability

Raw data generated and/or analyzed during the current study are available from the corresponding author on reasonable request.

## References

[1] Wang, Y. Y., Hsu, P. K. & Tsay, Y. F. Uptake, allocation and signaling of nitrate. *Trends Plant Sci.* **17** (8), 458–467. 10.1016/j.tplants.2012.04.006 (2012). 22658680 10.1016/j.tplants.2012.04.006

[2] Zhao, L., Liu, F., Crawford, N. M. & Wang, Y. Molecular regulation of nitrate responses in plants. *Int. J. Mol. Sci.* **19** (7), 2039. 10.3390/ijms19072039 (2018). 30011829 10.3390/ijms19072039 PMC6073361

[3] Vidal, E. A. et al. Nitrate in 2020: thirty years from transport to signaling networks. *Plant. Cell.* **32**, 2094–2119. 10.1105/tpc.19.00748 (2020). 32169959 10.1105/tpc.19.00748 PMC7346567

[4] Anas, M. et al. Fate of nitrogen in agriculture and environment: agronomic, eco-physiological and molecular approaches to improve nitrogen use efficiency. *Biol. Res.* **53**, 1–20. 10.1186/s40659-020-00312-4 (2020). 33066819 10.1186/s40659-020-00312-4 PMC7565752

[5] Loreti, E. & Perata, P. The many facets of hypoxia in plants. *Plants* **9** (6), 745. 10.3390/plants9060745 (2020). 32545707 10.3390/plants9060745 PMC7356549

[6] Kolozs, H. et al. Growth responses and adventitious root formation of cucumber hybrid lines in a waterlogged condition. *Horticulturae* **9** (10), 1102. 10.3390/horticulturae9101102 (2023).

[7] Kołton, A., Kęska, K. & Czernicka, M. Selection of tomato and cucumber accessions for waterlogging sensitivity through morpho-physiological assessment at an early vegetative stage. *Agronomy* **10**, 10. 10.3390/agronomy10101490 (2020).

[8] Olorunwa, O. J. et al. Short waterlogging events differently affect morphology and photosynthesis of two cucumber (*Cucumis sativus* L.) cultivars. *Front. Plant Sci.* **13**, 896244. 10.3389/fpls.2022.896244 (2022). 35937378 10.3389/fpls.2022.896244 PMC9355484

[9] Bramley, H. & Tyerman, S. Root water transport under waterlogged conditions and the roles of Aquaporins. In *Waterlogging Signalling and Tolerance in Plants* (eds. Mancuso, S. & Shabala, S.) 151–180 (Springer, 2010). 10.1007/978-3-642-10305-6_8.

[10] Parent, C., Capelli, N., Berger, A., Crèvecoeur, M. & Dat, J. F. An overview of plant responses to soil waterlogging. *Plant. Stress*. **2** (1), 20–27 (2008).

[11] Pan, J., Sharif, R., Xu, X. & Chen, X. Mechanisms of waterlogging tolerance in plants: research progress and prospects. *Front. Plant Sci.* **11**, 627331. 10.3389/fpls.2020.627331 (2021). 33643336 10.3389/fpls.2020.627331 PMC7902513

[12] van Dongen, J. T. & Licausi, F. Oxygen sensing and signaling. *Annu. Rev. Plant Biol.* **66**, 345–367. 10.1146/annurev-arplant-043014-114813 (2015). 25580837 10.1146/annurev-arplant-043014-114813

[13] Pucciariello, C. & Perata, P. The oxidative paradox in low oxygen stress in plants. *Antioxidants* **10** (2), 332. 10.3390/antiox10020332 (2021). 33672303 10.3390/antiox10020332 PMC7926446

[14] Voesenek, L. A. & Bailey-Serres, J. Flood adaptive traits and processes: an overview. *New Phytol.* **206** (1), 57–73. 10.1111/nph.13209 (2015). 25580769 10.1111/nph.13209

[15] Gupta, K. J. et al. The role of nitrite and nitric oxide under low oxygen conditions in plants. *New Phytol.* **225** (3), 1143–1151. 10.1111/nph.15969 (2020). 31144317 10.1111/nph.15969

[16] Timilsina, A., Dong, W., Hasanuzzaman, M., Liu, B. & Hu, C. Nitrate–nitrite–nitric oxide pathway: A mechanism of hypoxia and anoxia tolerance in plants. *Int. J. Mol. Sci.* **23** (19), 11522. 10.3390/ijms231911522 (2022). 36232819 10.3390/ijms231911522 PMC9569746

[17] Zhou, X. et al. Nitric oxide, crosstalk with stress regulators and plant abiotic stress tolerance. *Plant Cell Rep.* **40** (8), 1395–1414. 10.1007/s00299-021-02705-5 (2021). 33974111 10.1007/s00299-021-02705-5

[18] Kolbert, Z., Szőllősi, R., Feigl, G., Kónya, Z. & Rónavári, A. Nitric oxide signalling in plant nanobiology: current status and perspectives. *J. Exp. Bot.* **72** (3), 928–940. 10.1093/jxb/eraa470 (2021). 33053152 10.1093/jxb/eraa470

[19] Mur, L. A. et al. Nitric oxide in plants: an assessment of the current state of knowledge. *AoB Plants*. pls052. 10.1093/aobpla/pls052 (2013). 5. 10.1093/aobpla/pls052 PMC3560241 23372921

[20] Chamizo-Ampudia, A., Sanz-Luque, E., Llamas, A., Galvan, A. & Fernandez, E. Nitrate reductase regulates plant nitric oxide homeostasis. *Trends Plant Sci.* **22** (2), 163–174. 10.1016/j.tplants.2016.12.001 (2017). 28065651 10.1016/j.tplants.2016.12.001

[21] Timilsina, A. et al. Potential pathway of nitrous oxide formation in plants. *Fronti. Plant Sci.* 11. 1177 Doi: 10.3389%2Ffpls.2020.01177 (2020). 10.3389/fpls.2020.01177 PMC7412978 32849729

[22] Hill, R. D., Igamberdiev, A. U. & Stasolla, C. Preserving root stem cell functionality under low oxygen stress: the role of nitric oxide and phytoglobins. *Planta* **258**(5), 89. 10.1007/s00425-023-04246-5 (2023). 10.1007/s00425-023-04246-5 37759033

[23] Sánchez-Vicente, I., Fernández-Espinosa, M. G. & Lorenzo, O. Nitric oxide molecular targets: reprogramming plant development upon stress. *J. Exp. Bot.* **70** (17), 4441–4460. 10.1093/jxb/erz339 (2019). 31327004 10.1093/jxb/erz339 PMC6736187

[24] Millar, A. H. & Day, D. A. Nitric oxide inhibits the cytochrome oxidase but not the alternative oxidase of plant mitochondria. *FEBS Lett.* **398** (2-3), 155–158. 10.1016/s0014-5793(96)01230-6 (1996). 8977097 10.1016/s0014-5793(96)01230-6

[25] Piccinini, L., Nirina Ramamonjy, F. & Ursache, R. Imaging plant cell walls using fluorescent stains: the beauty is in the details. *J. Microsc.* **295**, 102–120. 10.1111/jmi.13289 (2024). 38477035 10.1111/jmi.13289

[26] Sasidharan, R. et al. Signal dynamics and interactions during flooding stress. *Plant Physiol.* **176** (2), 1106–1117. 10.1104/pp.17.01232 (2018). 29097391 10.1104/pp.17.01232 PMC5813540

[27] Miller, G., Shulaev, V. & Mittler, R. Reactive oxygen signaling and abiotic stress. *Physiol. Plant.* **133** (3), 481–489. 10.1111/j.1399-3054.2008.01090.x (2008). 18346071 10.1111/j.1399-3054.2008.01090.x

[28] Peláez-Vico, M. Á. et al. Rapid systemic responses of *Arabidopsis* to waterlogging stress. *Plant Physiol.* **193** (3), 2215–2231. 10.1093/plphys/kiad433 (2023). 37534775 10.1093/plphys/kiad433

[29] Tong, C. et al. Opportunities for improving waterlogging tolerance in cereal crops—physiological traits and genetic mechanisms. *Plants* **10**, 8. 10.3390/plants10081560 (2021). 10.3390/plants10081560 PMC8401455 34451605

[30] Frungillo, L., Skelly, M. J., Loake, G. J., Spoel, S. H. & Salgado, I. S-nitrosothiols regulate nitric oxide production and storage in plants through the nitrogen assimilation pathway. *Nat. Commun.* **5** (1), 5401. 10.1038/ncomms6401 (2014). 25384398 10.1038/ncomms6401 PMC4229994

[31] da-Silva, C. J. & do Amarante, L. Short-term nitrate supply decreases fermentation and oxidative stress caused by waterlogging in soybean plants. *Environ. Exp. Bot.* **176**, 36. 10.1071/fp22145 (2020).

[32] Krapp, A. et al. Nitrate transport and signalling in Arabidopsis. *J. Exp. Bot.* **65** (3), 789–798. 10.1093/jxb/eru001 (2014). 24532451 10.1093/jxb/eru001

[33] Fan, H. F., Du, C. X., Ding, L. & Xu, Y. L. Exogenous nitric oxide promotes waterlogging tolerance as related to the activities of antioxidant enzymes in cucumber seedlings. *Russ. J. Plant Physiol.* **61**, 366–373. 10.7868/S001533031403004X (2014).

[34] Ueda, Y., Konishi, M. & Yanagisawa, S. Molecular basis of the nitrogen response in plants. *Soil. Sci. Plant. Nutr.* **63** (4), 329–341. 10.1080/00380768.2017.1360128 (2017).

[35] Samant, S. B. et al. Nitric oxide, energy and redox-dependent responses to hypoxia. *J. Exp. Bot.* **2024**, erae139. 10.1093/jxb/erae139 (2024). 10.1093/jxb/erae139 38557811

[36] Gibberd, M. R., Gray, J. D., Cocks, P. S. & Colmer, T. D. Waterlogging tolerance among a diverse range of *Trifolium* accessions is related to root porosity, lateral root formation and ‘aerotropic rooting’. *Ann. Botany*. **88** (4), 579–589. 10.1006/anbo.2001.1506 (2001).

[37] Chen, T., Yuan, F., Song, J. & Wang, B. Nitric oxide participates in waterlogging tolerance through enhanced adventitious root formation in the euhalophyte *Suaeda Salsa*. *Funct. Plant Biol.* **43** (3), 244–253. 10.1071/fp15120 (2016). 32480457 10.1071/FP15120

[38] Niu, L. et al. Proteomic investigation of S-nitrosylated proteins during NO-induced adventitious rooting of cucumber. *Int. J. Mol. Sci.* **20**, 69. 10.3390/ijms20215363 (2019). 10.3390/ijms20215363 PMC6862188 31661878

[39] Li, G., Tillard, P., Gojon, A. & Maurel, C. Dual regulation of root hydraulic conductivity and plasma membrane Aquaporins by plant nitrate accumulation and high-affinity nitrate transporter NRT2. 1. *Plant Cell Physiol.* **57** (4), 733–742. 10.1093/pcp/pcw022 (2016). 26823528 10.1093/pcp/pcw022

[40] Tyerman, S. D., Wignes, J. A. & Kaiser, B. N. Root hydraulic and Aquaporin responses to N availability. In *Plant Aquaporins: from Transport To Signaling* (eds. Chaumont, F. & Tyerman, S. D.) 207–236 (Springer, 2017). 10.1007/978-3-319-49395-4_10.

[41] Pou, A. et al. Exposure to high nitrogen triggered a genotype-dependent modulation of cell and root hydraulics, which can involve aquaporin regulation. *Physiol. Plant.* **174**(1), e13640. 10.1111/ppl.13640 (2022). 10.1111/ppl.13640 35099809

[42] Gibbs, D. J. et al. Nitric oxide sensing in plants is mediated by proteolytic control of group VII ERF transcription factors. *Mol. Cell*. **53** (3), 369–379. 10.1016/j.molcel.2013.12.020 (2014). 24462115 10.1016/j.molcel.2013.12.020 PMC3969242

[43] Schmidt, R. R. et al. Low-oxygen response is triggered by an ATP-dependent shift in oleoyl-CoA in Arabidopsis. *Proc. Natl. Acad. Sci.* **115**(51), E12101-E12110. 10.1073/pnas.1809429115 (2018). 10.1073/pnas.1809429115 PMC6304976 30509981

[44] Gupta, K. J. et al. Nitric oxide regulation of plant metabolism. *Mol. Plant*. **15** (2), 228–242. 10.1016/j.molp.2021.12.012 (2022). 34971792 10.1016/j.molp.2021.12.012

[45] Nejamkin, A., Del Castello, F., Lamattina, L., Correa-Aragunde, N. & Foresi, N. Nitric oxide is required for primary nitrate response in arabidopsis: evidence for S-nitrosation of NLP7. *Antioxid. Redox. Signal.*10.1089/ars.2022.0210 (2023). 37597195 10.1089/ars.2022.0210

[46] Buet, A., Galatro, A., Ramos-Artuso, F. & Simontacchi, M. Nitric oxide and plant mineral nutrition: current knowledge. *J. Exp. Bot.* **70** (17), 4461–4476. 10.1093/jxb/erz129 (2019). 30903155 10.1093/jxb/erz129

[47] Li, H. et al. Nitric oxide generated by *Piriformospora indica*-induced nitrate reductase promotes tobacco growth by regulating root architecture and ammonium and nitrate transporter gene expression. *J. Plant Interact.* **17** (1), 861–872. 10.1080/17429145.2022.2108926 (2022).

[48] Sun, H. et al. Nitric oxide generated by nitrate reductase increases nitrogen uptake capacity by inducing lateral root formation and inorganic nitrogen uptake under partial nitrate nutrition in rice. *J. Exp. Bot.* **66** (9), 2449–2459. 10.1093/jxb/erv030 (2015). 25784715 10.1093/jxb/erv030 PMC4986861

[49] Dong, F., Simon, J., Rienks, M., Lindermayr, C. & Rennenberg, H. Effects of rhizopheric nitric oxide (NO) on N uptake in *Fagus sylvatica* seedlings depend on soil CO_2_ concentration, soil N availability and N source. *Tree Physiol.* **35** (8), 910–920. 10.1093/treephys/tpv051 (2015). 26093371 10.1093/treephys/tpv051

[50] Simon, J., Dong, F., Buegger, F. & Rennenberg, H. Rhizospheric NO affects N uptake and metabolism in Scots pine (*Pinus sylvestris* L.) seedlings depending on soil N availability and N source. *Plant. Cell. Environ.* **36** (5), 1019–1026. 10.1111/pce.12034 (2013). 23146102 10.1111/pce.12034

[51] Camut, L. et al. Nitrate signaling promotes plant growth by upregulating Gibberellin biosynthesis and destabilization of DELLA proteins. *Curr. Biol.* **31** (22), 4971–4982. 10.1016/j.cub.2021.09.024 (2021). 34614391 10.1016/j.cub.2021.09.024

[52] Guan, P. Dancing with hormones: a current perspective of nitrate signaling and regulation in Arabidopsis. *Front. Plant Sci.* **8**, 36. 10.3389/fpls.2017.01697 (2017). 10.3389/fpls.2017.01697 PMC5625010 29033968

[53] Vidal, A., Cantabella, D., Bernal-Vicente, A., Díaz-Vivancos, P. & Hernández, J. A. Nitrate-and nitric oxide-induced plant growth in pea seedlings is linked to antioxidative metabolism and the ABA/GA balance. *J. Plant Physiol.* **230**, 13–20. 10.1016/j.jplph.2018.08.003 (2018). 30138843 10.1016/j.jplph.2018.08.003

[54] Sasidharan, R., Schippers, J. H. & Schmidt, R. R. Redox and low-oxygen stress: signal integration and interplay. *Plant Physiol.* **186** (1), 66–78. 10.1093/plphys/kiaa081 (2021). 33793937 10.1093/plphys/kiaa081 PMC8154046

[55] Kocsy, G. et al. Redox control of plant growth and development. *Plant Sci.* **211**, 77–91. 10.1016/j.plantsci.2013.07.004 (2013). 23987814 10.1016/j.plantsci.2013.07.004

[56] Mosa, K. A. et al. Copper nanoparticles induced genotoxicty, oxidative stress, and changes in superoxide dismutase (SOD) gene expression in cucumber (*Cucumis sativus*) plants. *Front. Plant. Sci.* **9**10.3389/fpls.2018.00872 (2018). 10.3389/fpls.2018.00872 PMC6055047 30061904

[57] Hodges, D., DeLong, J., Forney, C. & Prange, R. K. Improving the thiobarbituric acid-reactive-substances assay for estimating lipid peroxidation in plant tissues containing anthocyanin and other interfering compounds. *Planta* **207**, 604–611. 10.1007/s004250050524 (1999). 10.1007/s00425-017-2699-3 28456836

[58] Mostofa, M. G., Fujita, M. & Tran, L. S. P. Nitric oxide mediates hydrogen peroxide- and Salicylic acid-induced salt tolerance in rice (*Oryza sativa* L.) seedlings. *Plant. Growth Regul.* **77**, 265–277. 10.1007/s10725-015-0061-y (2015).

[59] ISO, International Organization for Standardization. Fruits, vegetables and derived products – Determination of nitrite and nitrate content. ISO 6635 (1984).

[60] Hesari, N. et al. High-nitrate-supply-induced transcriptional upregulation of ascorbic acid biosynthetic and recycling pathways in cucumber. *Plants Doi*. 10.3390/plants12061292 (2023). 10.3390/plants12061292 PMC10051573 36986979

[61] Kim, J. Y. & Seo, H. S. In vitro nitrate reductase activity assay from Arabidopsis crude extracts. *Bio-Protocol* **8**, e2785. 10.21769/BioProtoc.2785 (2018). 34286008 10.21769/BioProtoc.2785 PMC8275288

[62] Kang, Y. Y., Guo, S. R., Li, J. & Duan, J. J. Effect of root applied 24-epibrassinolide on carbohydrate status and fermentative enzyme activities in cucumber (*Cucumis sativus* L.) seedlings under hypoxia. *Plant. Growth Regul.* **57**, 259–269. 10.1007/s10725-008-9344-x (2009).

[63] Jaakola, L., Pirttilä, A. M., Halonen, M. & Hohtola, A. Isolation of high quality RNA from Bilberry (*Vaccinium myrtillus* L.) fruit. *Mol. Biotechnol.* **19**, 201–203. 10.1385/MB:19:2 (2001). 11725489 10.1385/MB:19:2:201

[64] Migocka, M., Warzybok, A. & Kłobus, G. The genomic organization and transcriptional pattern of genes encoding nitrate transporters 1 (NRT1) in cucumber. *Plant. Soil.* **364**, 245–260. 10.1007/s11104-012-1345-x (2013).

[65] Oszlányi, R. et al. Oxidative stress level and dehydrin gene expression pattern differentiate two contrasting cucumber F1 hybrids under high fertigation treatment. *Int. J. Biol. Macromol.* **161**, 864–874. 10.1016/j.ijbiomac.2020.06.050 (2020). 32535210 10.1016/j.ijbiomac.2020.06.050

[66] Szegő, A. et al. Downregulation of polyamine and Diamine oxidases in Silicon-Treated cucumber. *Plants* **10**, 56. 10.3390/plants10061248 (2021). 10.3390/plants10061248 PMC8235019 34205296

[67] Kolbert, Z. et al. S-Nitrosothiol signaling is involved in regulating hydrogen peroxide metabolism of zinc-stressed Arabidopsis. *Plant. Cell. Physiol.* **60**, 2449–2463. 10.1093/pcp/pcz138 (2019). 31340034 10.1093/pcp/pcz138

[68] Laemmli, U. Cleavage of structural proteins during the assembly of the head of bacteriophage T4. *Nature* **227**, 680–685. 10.1038/227680a0 (1970). 5432063 10.1038/227680a0

[69] Nakazawa, M. Fmsb: functions for medical statistics book with some demographic data (2021, accessed 27 May 2024). https://cran.r-project.org/package=fmsb .

[70] Lê, S., Josse, J. & Husson, F. FactoMineR: an R package for multivariate analysis. *J. Stat. Softw.* **25**, 1–18 (2008).

[71] Wickham, H. *Ggplot2: Elegant Graphics for Data Analysis* (Springer, 2016). 10.1007/978-3-319-24277-4.

[72] Kassambara, A. & Mundt, F. Package ‘factoextra’. Extract visualize results multivar. *Data Analyses* **2017**, 76 (2017).

