# Supplementary material for: Nitric oxide mediates nitrate induced alleviation of waterlogging stress in cucumber
Source: Sci Rep. 2025 May 1;15:15307. doi: 10.1038/s41598-025-00321-x (PMC12045955; doi:10.1038/s41598-025-00321-x)

Table S1. Composition of the applied half strength Hoagland fertigation solution

**Macronutrient**	**Concentration**
**KNO_3**	2.5 mM
**$Ca(NO_3)_2$**	2.5 mM
**$MgSO_4$**	1.0 mM
**KH_2PO_4**	0.5 mM
**Micronutrient**	
**H_3BO_3**	0.1 µM
**Na_2MoO_4**	5.0 µM
**$ZnSO_4$**	0.5 µM
**$MnCl_2$**	1.0 µM
**$CuSO_4$**	0.25 µM
**$CoCl_2$**	0.1 µM
**$NiSO_4$**	0.1 µM
**NaFe-EDTA**	5 µM
**MES Buffer**	0.5 mM

Table S2. List of the target genes and oligonucleotide primers for RT–PCR and qRT–PCR analysis.

**Gene**	**Accession No**	**Forward primer (5'-3')**	**Reverse primer (5'-3')**	**bp** cDNA	**bp** gDNA
CsNRT1.5a	CsaV3_3G010210	ATATCGGCGACCGATAAC	ACTACACTGGCAACTACAC	230	230
CsNRT1.8	CsaV3_3G001980	CCTTGCCCTGAACCTTGGAT	CTCATCGGAACTCCGCGTTA	144	144
CsRBOH3	CsaV3_6G021970	CCTGACGATGGGATTACTCT	ACCATACTGAAGTGGACTGC	161	625
CsRBOH4	CsaV3_1G002910	GCTCTAGTCTTGCTTCCTGT	CATGGAGGAACTCATCTTGC	161	623
CsRBOH6	CsaV3_5G002350	GCTTGGAACGAAATACAGAG C	CTCAAAGCATCGAACAGTTCC	131	244
CsRBOH9	CsaV3_1G038860	CGTGCATTTCGATCAGGCTA	CCAATCTCCTACAGTGCGAA	147	437
CsGSNOR	CsaV3_6G007280	GGCAAAAATTGATCCCACGG	TCAGTCATTGTTGGGGTTGC	388	3838
CsRAP2.3	CsaV3_6G022740	CCACGAAAAGGAGTTCGAG	CCGGTAAAAATGCAGTGCG	172	172
CsHem3	CsaV3_2G031100	GCAAGATAAGGCTTCCG	GGAATTCGAAGAAGGAGG	185	185
CsActin	CsaV3_6G041900	TTCTGGTGATGGTGTGAGTC	CATGTTCACCACCACTGCC	151	151

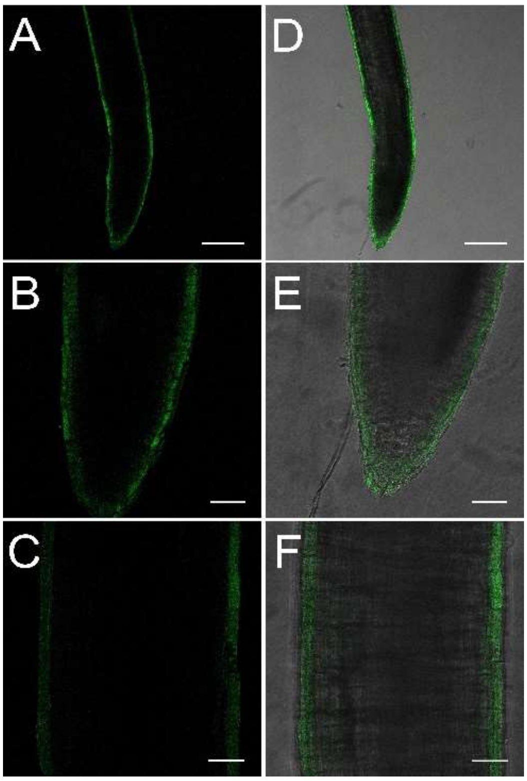

Figure S1 Autofluorescence detected in the outermost cell layer of root caps (A,D; B,E) and in the exodermal cells of the differentiation zone (A,D; C,F) in non-labelled, non-treated cucumber roots (representative images). The excitation range overlapped with that of DAF-FM DA. Note that except for these cell types, no autofluorescence was detected either in the cell division and transition (A,D; B,E) or in the differentiation (A,D; C,F) zones. A, B, C: fluorescent images, D, E, F: merged images. Bar = 250 µm (A, D); 50 µm (B, C, E, F).

Uncropped gel images for Figure 12

Full gel images of some of the many biological and technical replicates of the RT-PCR results that have been used to create Figure 12 are provided here.

Root:
RBOH4,

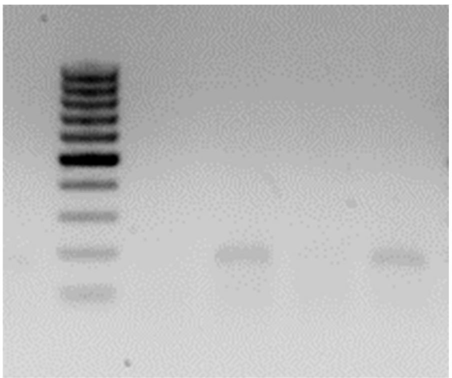

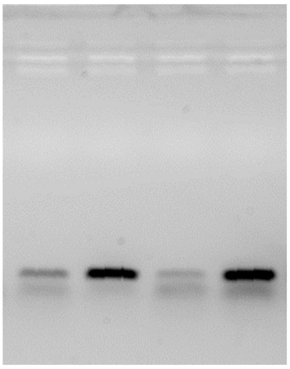

RBOH6,

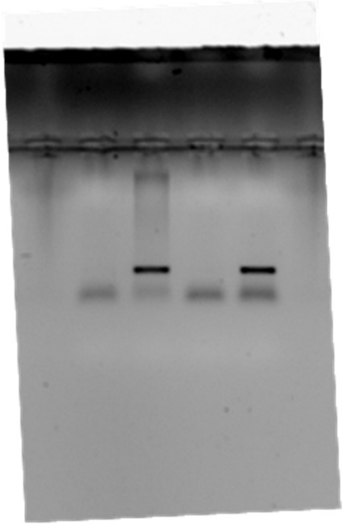

RBOH9,

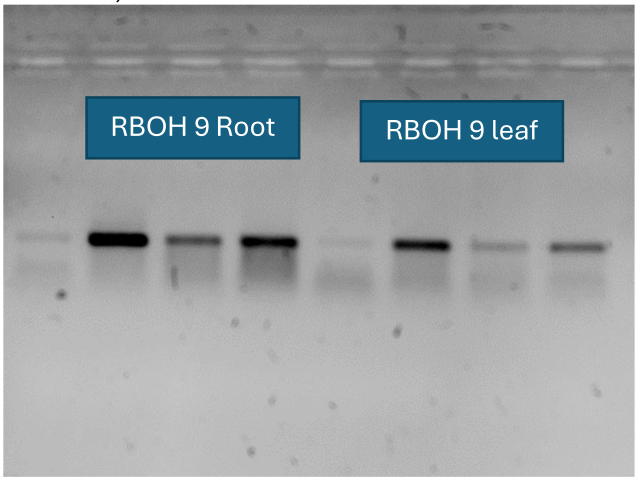

GSNOR,

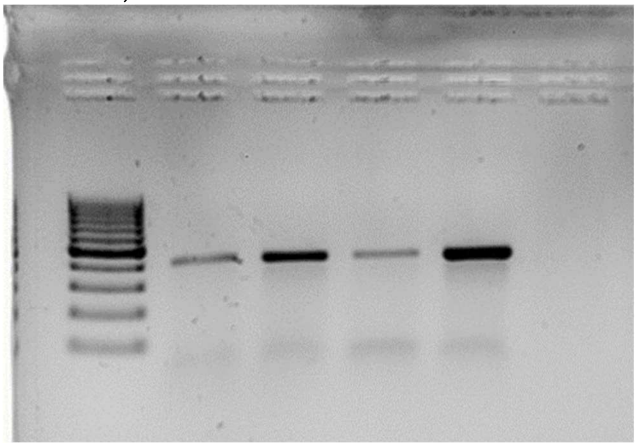

RAP2.3, and Hem3,

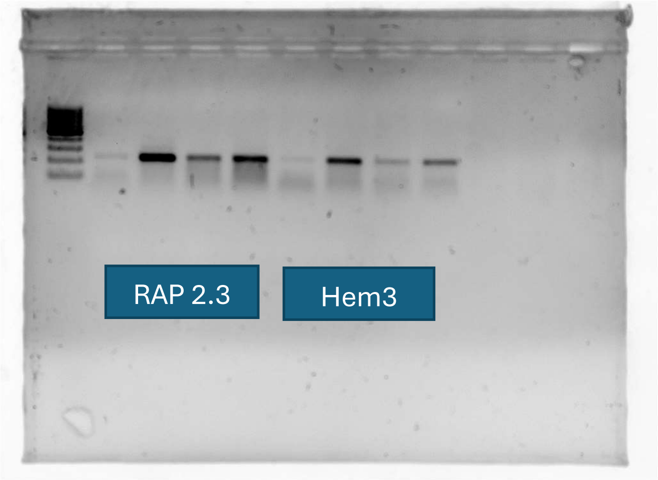

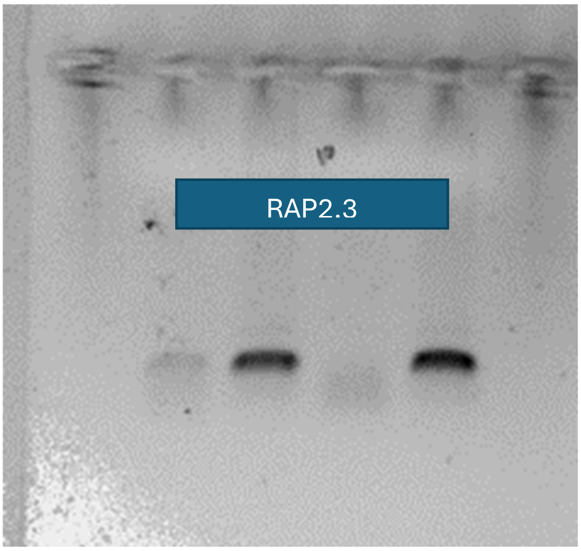

NRT1.3,

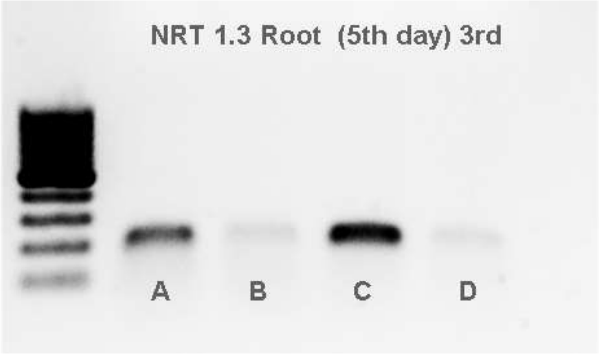

NRT1.5,

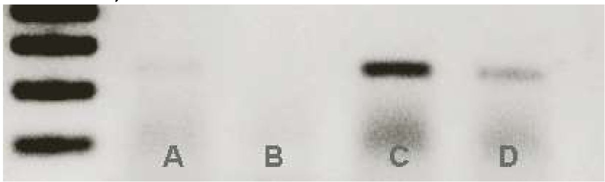

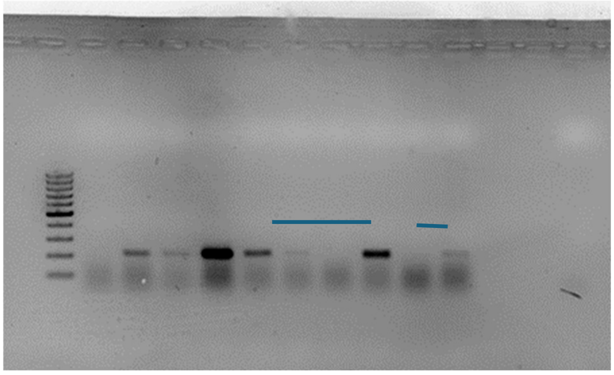

extra lane, results not included

NRT1.8

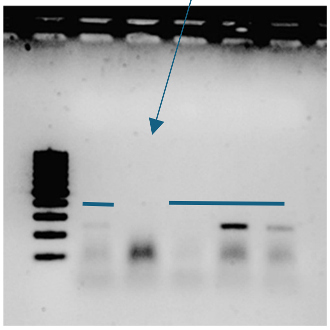

Actin

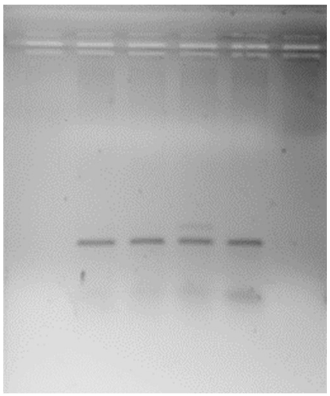

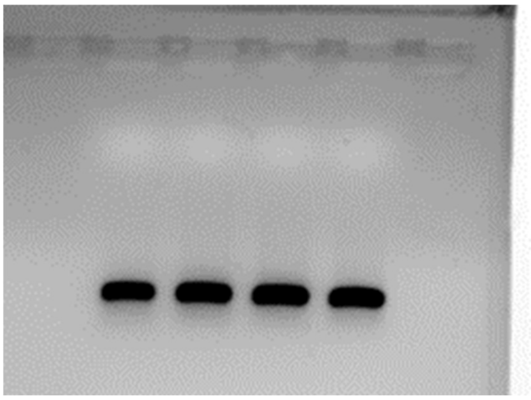

Leaf

RBOH6,

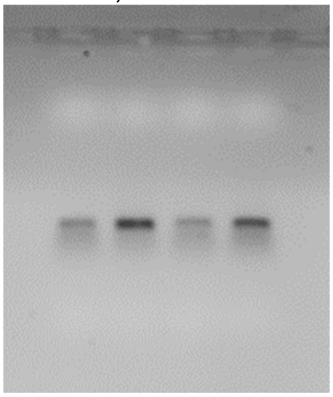

RBOH9,

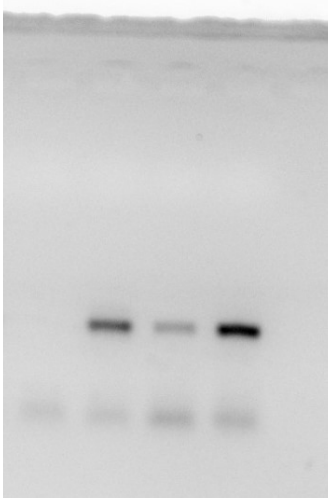

Actin

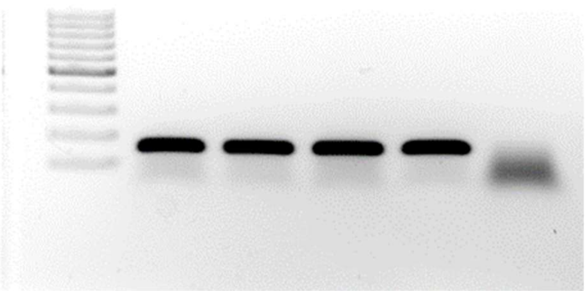

Supplement: Supplementary file 1 — Supplementary Material 1 [file 41598_2025_321_MOESM1_ESM.pdf]
